# HIV-1 Tropism Dynamics and Phylogenetic Analysis from Longitudinal Ultra-Deep Sequencing Data of CCR5- and CXCR4-Using Variants

**DOI:** 10.1371/journal.pone.0102857

**Published:** 2014-07-17

**Authors:** Mariano M. Sede, Franco A. Moretti, Natalia L. Laufer, Leandro R. Jones, Jorge F. Quarleri

**Affiliations:** 1 Instituto de Investigaciones Biomédicas en Retrovirus y Sida (INBIRS), Universidad de Buenos Aires, CONICET, Buenos Aires, Argentina; 2 Consejo de Investigaciones Científicas y Técnicas (CONICET), Buenos Aires, Argentina; 3 Laboratorio de Virología y Genética Molecular, Facultad de Ciencias Naturales, sede Trelew, Universidad Nacional de la Patagonia San Juan Bosco, Chubut, Argentina; University of Sydney, Australia

## Abstract

**Objective:**

Coreceptor switch from CCR5 to CXCR4 is associated with HIV disease progression. The molecular and evolutionary mechanisms underlying the CCR5 to CXCR4 switch are the focus of intense recent research. We studied the HIV-1 tropism dynamics in relation to coreceptor usage, the nature of quasispecies from ultra deep sequencing (UDPS) data and their phylogenetic relationships.

**Methods:**

Here, we characterized C2-V3-C3 sequences of HIV obtained from 19 patients followed up for 54 to 114 months using UDPS, with further genotyping and phylogenetic analysis for coreceptor usage. HIV quasispecies diversity and variability as well as HIV plasma viral load were measured longitudinally and their relationship with the HIV coreceptor usage was analyzed. The longitudinal UDPS data were submitted to phylogenetic analysis and sampling times and coreceptor usage were mapped onto the trees obtained.

**Results:**

Although a temporal viral genetic structuring was evident, the persistence of several viral lineages evolving independently along the infection was statistically supported, indicating a complex scenario for the evolution of viral quasispecies. HIV X4-using variants were present in most of our patients, exhibiting a dissimilar inter- and intra-patient predominance as the component of quasispecies even on antiretroviral therapy. The viral populations from some of the patients studied displayed evidences of the evolution of X4 variants through fitness valleys, whereas for other patients the data favored a gradual mode of emergence.

**Conclusions:**

CXCR4 usage can emerge independently, in multiple lineages, along the course of HIV infection. The mode of emergence, *i.e.* gradual or through fitness valleys seems to depend on both virus and patient factors. Furthermore, our analyses suggest that, besides becoming dominant after population-level switches, minor proportions of X4 viruses might exist along the infection, perhaps even at early stages of it. The fate of these minor variants might depend on both viral and host factors.

## Introduction

Human immunodeficiency virus type 1 (HIV-1) entry into host cells requires synchronized interactions of the envelope glycoprotein gp120 with the CD4 receptor and with one of the chemokine receptors, CCR5 or CXCR4. HIV-1 tropism for the chemokine receptors CCR5 and CXCR4 has been shown to be associated with disease progression [1]. Viruses, especially those using the CCR5 receptor to enter the target cells (R5 viruses), are generally predominant at early stages of HIV-1 infection, whereas the emergence of CXCR4-using viruses (X4 viruses) generally occurs at later stages [2]. Variation in R5 Env proteins can also influence the ability of a virus to utilize various levels of CD4 and CCR5 found in different cell types, such as macrophages and T-cells. The presence of X4 viruses is consistently associated with low CD4+ T-cell counts and accelerated disease progression, although it is still unclear whether it is the cause or consequence of disease progression [3], [4]. Inferring HIV-1 coreceptor usage from a genotype is becoming more and more important for appropriately treating long-term patients as the level of CXCR4-using viruses is associated with risk of virological failure to maraviroc-containing regimens in a dose-dependent fashion [5], [6].

The primary genetic determinant for the HIV coreceptor usage is the third variable region (V3) of the HIV gp120 envelope glycoprotein encompassing approximately 35 residues (varying in length depending on the viral isolate) with a conserved disulfide bridge at the base of the loop. The presence of basic residues at V3 reference positions 11 and 25 is strongly predictive of CXCR4 usage but there are exceptions to this rule. The evolution of CXCR4 use following a host-specific mutational pathway could be influenced by the immune pressure that promotes a rapid host-specific adaptation. This scenario appears to limit the incidence of the R5-to-X4 coreceptor switch.

The detection of HIV quasispecies at very low frequencies is limited when standard bulk-nucleic acid sequencing methods are used. The phenotypic assays to assess HIV tropism can reliably detect minorities of less than 5% of the viral population but are cost-intensive and time-consuming [7]. Taking into account the plausible emergence of HIV minor variants during the follow-up, our analysis involved the use of powerful technology of ultra-deep pyrosequencing (UDPS) after PCR amplification of the *env* gene, coding for the viral glycoprotein gp120, encompassing the tropism-related V3 loop region.

The aim of the present study was to analyze the HIV-1 tropism dynamics in relation to coreceptor usage, the nature of HIV-1 quasispecies and their phylogenetic relationships from a large nucleotide sequence data generated by UDPS, prior and during long term antiretroviral therapy.

The HIV tropism analysis of the vast amounts of sequence data was automated by computer assistance using the geno2pheno 454 system [5]. It handles all processing and prediction steps involved in the prediction of coreceptor usage from UDPS data. Such data together with their known sampling dates were used to establish phylogenetic relationships and to analyze the evolution model for the dynamics of HIV coreceptor usage.

## Materials and Methods

### Patients

A total of 133 stored plasma samples obtained from nineteen HAART (highly active antiretroviral therapy)-treated patients were collected yearly for a 6 to 11-yr period of follow-up. No patient received CCR5 antagonists. We selected HIV infected adults with well known anti-viral therapy histories and schedule, and a good adherence record. All patients were followed by a minimum of 6 years and sampled at least four times during the study. Their history of HIV infection was based on time from seroconversion. Baseline for drug-naive individuals was considered as the first time they were assessed at INBIRS, while it was the time of the first sample available for those HAART-experienced patients (Table 1).

**Table 1:** Demographic characteristics of the study population^1^.

	Group A HAARTresponders (n = 6)	Group B HAARTnon-responders (n = 13)
Age (years)	43.5 (±6.6)	45.3 (±6.4)
Male/female	4/2	11/2
HIV time post-infection^2^	20.2 (±5.7)	16.6 (±4.9)
Time of follow-up (months)	73 (±10)	79 (±22)
HIV subtype (B / non-B)	0/6	4/9
Time on HAART (years)	7.2 (±0.8)	11.6 (±4.2)
HIV viral load (pre-HAART/intra-HAART)^3^	4.66±0.61/<1.70	4.30±1.45/4.24±1.66
CD4 T cell count (pre-HAART/intra-HAART)^4^	262±80/357±237	460±243/324±147
%X4 using variants at baseline^5^	2.2 [0.5−56.9]	4.1 [0−17.8]

1 Mean (±standard deviation).

2 HIV infection date calculated as mid-point between last negative and first positive samples are indicated as years.

3 Expressed as log copies/ml.

4 Expressed as cells/µl.

5 Median [IQR].

CD4 T cell count and HIV-RNA plasma level (range of 50–500,000 HIV-1 RNA copies/mL; VERSANT HIV-1 RNA version 3.0 bDNA Assay, Siemens Diagnostics) were measured at sample collection. The optimal virological response was defined as having a viral load ≤50 copies/mL (≤1.7 log) at each measurement throughout the study. To detect HIV-1 viremia below this threshold with an input plasma volume of 500 µl, a centrifugation step prior to RNA extraction was added (23,500× g at 4°C for 60 min (Mikro22R, Hettich, Germany) and the dilution factors were decreased [8]. HIV genotypic tropism testing was performed using the stored plasma samples.

The research project was submitted to the Fundación Huésped Ethics Committee Review Board, Buenos Aires, on June 27th, 2012. It was approved on July 02, 2012. All participants recruited in the present study had been included in a larger, previous project. The latter involved clinical, virological and epidemiological aspects of the HIV/HCV coinfection and all patients provided an informed consent which was recorded during sample collection. Taking into consideration that the present study is centered on additional virological aspects of the HIV infection and that the biological samples used were those previously collected, there was no need for additional patient visits. Hence, the Fundación Huésped Ethics Committee Review Board waived the need for a new written informed consent from the participants for the present study. The design of the present study (retrospective and longitudinal) and the conditions detailed above were considered at the moment of requesting the Ethics Committee for the procedure approval.

### RNA extraction, cDNA synthesis and V3-nested PCR

RNA extraction was performed using the Viral RNA mini kit (Qiagen, Hilden, Germany) according to the manufacturer’s instructions. cDNA-synthesis was performed using 10 µl of RNA, specific primer V3-r (5′ GAGGGGAATTTTTCTACTGT, position 7572-7533 in HXB2), [9] and Superscript II (Invitrogen) according to the manufacturer’s instructions in a total volume of 20 µl.

Amplification of the V3 region was carried out using the specific primers V3-r and the V3-f (5′ CACAGTACAATGTACACATG, position 6943–6962 in HXB2) for the first round of the PCR followed by the nested reaction using the primers V3-nf (5′ AAATGGCAGTCTAGCAGAAG, position 7006–7025 in HXB2) and V3-nr (5′ ACAATTTCTGGGTCCCCTCC, position 7338–7319 in HXB2). The product size is 320 bp long. The primers employed for the second round were modified by 5′ Tag extensions, which provided binding sites for the multiplex identifiers (MIDs). The tags were as follows: sense-Tag 5′-CACGACGTTGTAAAACGA-3′; antisense-Tag 5′-CAGGAAACAGCTATGACC-3′. MIDs allowed for the identification of samples after the pyrosequencing procedure was complete.

In order to ensure a good sampling as well as to optimize the study of the genetic heterogeneity of viral population present in 0.75 ml of plasma by UDPS analysis, PCR amplicons from 3 reactions were pooled for each sample.

### Ultra-deep pyrosequencing (UDPS)

Ultra deep pyrosequencing (UDPS) on the Roche/454 Life Sciences “Genome Sequencer-FLX” (GS-FLX) is a sensitive sequencing technique able to detect low-frequency subpopulations of virus and to generate thousands of sequences from a given sample [10], [11].

After PCR amplification, PCR-purified amplicons were quantified using Quant-iT PicoGreen (Invitrogen, Life Technologies, MI, USA). In addition, an Agilent 2100 bioanalyzer (Agilent Life Science, Santa Clara, California, USA) was used to verify the quality and length of amplicons.

After quality controls, PCR amplicons were pooled in equimolar concentrations; subsequently PCR amplicons were combined at an appropriate ratio with DNA capture microbead. Emulsion PCR was performed, and DNA and beads were washed, purified and prepared for pyrosequencing according to the manufacturer’s instructions. HIV-V3 sequence and flanking genomic regions amplified on each bead were determined by pyrosequencing on the GS-FLX [12]. The raw sequence output (‘reads’) generated by Roche/454 GS-FLX platform was processed by a script that sorted reads by region and trimmed the tags.

There is a growing realization of the potential bias that sequencing errors can introduce in next generation sequencing data [13]. Here, we filtered the data obtained based on sequence length, quality of base calls, frame shifts and k-mer frequencies, and compared UDPS data with Sanger sequences obtained from molecular clones as described below.

In phylogenetic analyses we prioritize the detection of minor variants, which may represent intermediate genotypes, low frequency variants, or transient links among the prevalent haplotypes. The most prevalent sequencing errors in 454 data correspond to spurious insertions and deletions in homopolymeric sequence regions. These errors have minimal or no effect in probabilistic phylogenetic analysis, due to the lack of a framework for modeling nucleotide insertions/deletions together with nucleotide substitutions, and thus indels are either ignored or treated as missing/unknown characters by most maximum likelihood and Bayesian phylogenetic algorithms. Missing data are treated as any of A, T, C or G and therefore cannot support any particular phylogenetic hypothesis [14]. Therefore, the influence of indels in phylogenetic analysis is negligible compared to the impact that minor, real variants can have on character optimization and thus on phylogenetic inference. Here, we did pay much attention to large read length differences, as they can result in large stretches of missing data in sequence alignments, and consequently in inflated levels of uncertainty (e.g. large numbers of equally good character optimizations, excessive numbers of floating taxa, misleading bootstrap supports, etc.). Thus, we filtered out reads with lengths <300 bases. Pyrosequencing data are neither free of erroneous base calls, which present rates similar to Sanger sequencing ones [15]. We discarded all sequences displaying no base calls above 10, and for the remaining sequences, all base calls with qualities bellow 10 were replaced by Ns. Then, we trimmed out low quality read ends such that no sequence with more than 5% positions with Ns were kept in the processed dataset. After that, a second length filtering round was applied to the remaining reads. These procedures were performed using *ad-hoc* R scripts [16]. The sequences obtained were submitted to a first round of phylogenetic analysis as described below (section Phylogenetic analysis), after which ostensibly large terminal branches, which we attribute to outlier, possibly erroneous and/or chimera reads, were removed (Fig. S1).

Prior to the determination of viral tropism along the follow up (see section “HIV tropism prediction from genotype”), the data were further filtered out by the presence of frame shifts, and low frequency k-mers were corrected by the Kec program [17].

In addition to the in-silico filtering described above, the error rate of pyrosequencing analyzer was assessed by comparing UDPS data vs. data generated by molecular cloning (Table S1). Briefly, for this goal we have selected two patients (Pat1 and Pat3) with the corresponding 4 sampling times. From new aliquots of their plasma samples, PCR amplicons from 3 independent reactions were obtained and pooled following identical lab procedures as described for UDPS. Such product was further cloned, and 14–20 clones were Sanger sequenced. For each viral haplotype the nucleotide and amino acid sequence obtained by both strategies were compared qualitatively (identical nucleotide and amino acid sequences) and quantitatively (relative frequencies in the total population). The results were statistically analyzed on their relative frequencies and no significant differences were found, indicating that no major quantitative biases were introduced by the experimental or computational processing methods.

### HIV tropism prediction from genotype

The prediction of HIV coreceptor usage was carried out by genotypic methods taking into consideration the availability of timesaving, reliable and widely available algorithms [18].

Reads that passed the quality controls described above were input to Geno2pheno [coreceptor] application at www.genafor.org. This is a bioinformatic tool for HIV coreceptor prediction based on a genotype approach [19]. This algorithm was followed for each HIV viraemic plasma sample characterized during the follow-up. A false positive rate (FPR) of 10% was chosen for detecting X4-variants. As recommended by the European guidelines for HIV-1 tropism determination, the cut-off used in all the analyses carried out in this study, was chosen since it has been shown to be a good predictor in both multi-experienced and drug-naïve patients [20], [21]. By re-analyzing the MOTIVATE and A4001029 trials, tropism predictions using V3 genotyping were shown to be comparable, based on single or triplicate testing using a FPR of 10% [22], [23].

HIV-1 tropism was also predicted by two alternative algorithms. However, taking into account the magnitude of nucleotide sequence data emerged from UDPS, such models were applied only for those viral variants detected most frequently (≥5%) at each sampling time. For this goal both position-specific scoring matrix (PSSM) matrices (PSSM X4/R5 or PSSM SI/NSI) based on single V3 sequences under their original cut-off values [24], and the very recently described (SAAC+BLAST) hybrid’ approach (named HIVcoPred) fixing a threshold of 0.5 [25] were carried out.

### Phylogenetic analysis

Sequence alignments were generated by the parallel version of the MAFFT multiple sequence alignment program, with default *op* and *ep.* Iterative refinement, and the weighted sum-of-pair scores and consistency score obtained from local alignments [26] were used for individual phylogenies, whereas the alignment of the reads from all patients was performed with the *PartTree* algorithm [27]. The datasets obtained were considerably large. Individual datasets ranged from 161 sequences in patient 23 to 2549 sequences in patient 6, whereas the full dataset (*i.e.* the sequence alignment containing the sequences from all the 19 patients) had 24335 entries. The inference of optimal trees from large datasets is a problem that cannot be solved in polynomial time [28], [29], [30]. However, heuristic methods have been developed that allow exploring tree spaces with reasonable intensities and in affordable times [31], [32], [33]. Here, phylogenetic trees were inferred by the FastTree 2 program [34]. This program combines minimum-evolution subtree-pruning-regrafting (SPR) and maximum likelihood nearest-neighbor interchange (NNI) tree searches. FastTree searches were performed with default parameters, which scale search intensity according to dataset sizes by using up to 4xlog_2_(*N*) rounds of minimum-evolution NNI, 2 rounds of SPR moves and up to 2xlog(*N*) rounds of maximum-likelihood NNIs, where *N* is the number of unique sequences in the dataset. Branch supports were calculated using the phylogenetic bootstrap. For these analyses, 100 resampled datasets were generated with the *Seqboot* component of the *Phylip* package *v6.3* [35] and analyzed by FastTree *2* and the *CompareToBootstrap.pl* module provided with the FastTree distribution. Given the large size of some of the phylogenetic trees obtained, Kec haplotypes [section Ultra-deep pyrosequencing (UDPS)] were also submitted for phylogenetic analysis to present a clearer evolutionary relationship of these variants.

For hypothesis testing, the analyses were constrained by allowing only trees congruent with each hypothesis and the trees obtained were compared against the corresponding unconstrained trees. To test for the persistence of viral lineages along the course of infection, searches were constrained such that sequences clustered according to sampling time. Additionally, the idea that X4 tropism evolves by traversing across low fitness valleys was also tested [36]. Should this hypothesis be correct, the occurrence of stochastic tunneling processes must be expected resulting in X4 sequences clustered into monophyletic groups [37]. Thus, for patient trees that displayed clades composed of X4 sequences mixed with small amounts of R5 ones, tree searches were constrained so that these R5 sequences were excluded from these clades. Significance tests were performed using both the approximately unbiased (AU) and o non-scaled bootstrap probability (NP) tests of CONSEL [38].

### HIV Quasispecies Heterogeneity Analysis based on V3 loop amino acid sequences

UDPS sequences resulting from the correction pipeline were analyzed to assess intra-patient diversity and quasispecies complexity dynamics during follow-up. To assess diversity, the mean genetic distance of amino acid sequences was calculated by PROTDIST using Jones-Taylor-Thornton matrix [http://caps.ncbs.res.in/iws/protdist.html].

To study the frequency distribution of reads in a viral population rather than the total number of reads, we computed the Shannon entropy. It has been defined in terms of the probabilities of the different sequences than can appear at a given time-point. This measure was calculated as -∑ p(i) log_2_ p(i) where p(i) is the relative frequency of each read i (i = 1,…n, where n is the total number of groups of identical reads) [39]. This quantity measures the amount of uncertainty in the distribution and was used in a similar manner by Delwart et al. [40]. The resulting number was normalized as a function of the number of clones analyzed, thus allowing comparisons of complexity among different isolates. The normalized entropy, Sn, was calculated as Sn = S/log_2_N, where N is the total number of sequences analyzed. Sn is 0 when there is a single read (i.e., n = 1, no diversity) and reaches its maximum value when the reads observed are equally frequent [i.e., p(i) = 1/n for all i]. We monitored the changes in the distribution of reads in an individual via determining the entropy and looked for changes in this value.

### TSL Matrix Based Input Vectors

TSL is an online tool (http://www.twosamplelogo.org/cgi-bin/tsl/tsl.cgi) which distinguishes the residue frequency between two types of datasets, on each of the positions of the given sample sequences. In addition to generating a graphical representation of the two given datasets (Positive and Negative sample), it also generates the output format as TXT (raw values) which is the residues’ frequency difference in the two samples with significance value (as shown by p-value). This table with position-specific frequency value was used to generate the frequency score of residues in CCR5 and CXCR4 sequences independently. Since each V3 peptide was 35 amino acids long, an input vector of 35 dimensions was generated.

### Statistical analysis

Baseline characteristics of the study population were recorded as absolute numbers and percentages and means ± SD for qualitative and quantitative variables, respectively. Mann-Whitney U testing was used for group comparison purposes. The association among quantitative variables was tested using the Spearman correlation coefficient. Statistical significance was assumed for P values below 0.05. All statistical analyses were performed using SPSS v15.0 (SPSS Inc., Chicago, IL, USA).

## Results

### Patient’s characteristics

The population was comprised of 15 males (44.8±5.6 years old) and 4 females (42.4±8.1 years old). Laboratory parameters were recorded longitudinally (Table S2). Thirteen patients (Pat1, 3, 5, 6, 7, 8, 9, 18, 24, 25, 26, 27, and 28) were drug-naive individuals who initiated antiretroviral therapy and have been on regular follow-up since then. The remaining six had a previous history of HAART exposure. These HIV infected persons have attended the Instituto de Inverstigaciones Biomédicas en Retrovirus y Sida (INBIRS) from 2002. They could be differentiated based on HAART response during the entire follow-up: (A) Subjects achieving sustained optimal virological responses to HAART with undetectable HIV-1 RNA for a mean of 7.2 years (patients responding to HAART; n = 6); and (B) Subjects exhibiting at least one episode of detectable HIV-1 RNA (HAART-treatment failure; n = 13) (Table 1).

### Comparison between HIV viral load and CD4 T cell count according to HAART response. Detection of Predicted X4 and R5-Using V3 Sequences by Ultra-Deep Sequencing

Of the 19 HIV infected patients involved in this longitudinal study, 133 plasma samples were yearly collected. These individuals were followed up for 76.4±18.4 months.

**Figure 1:**
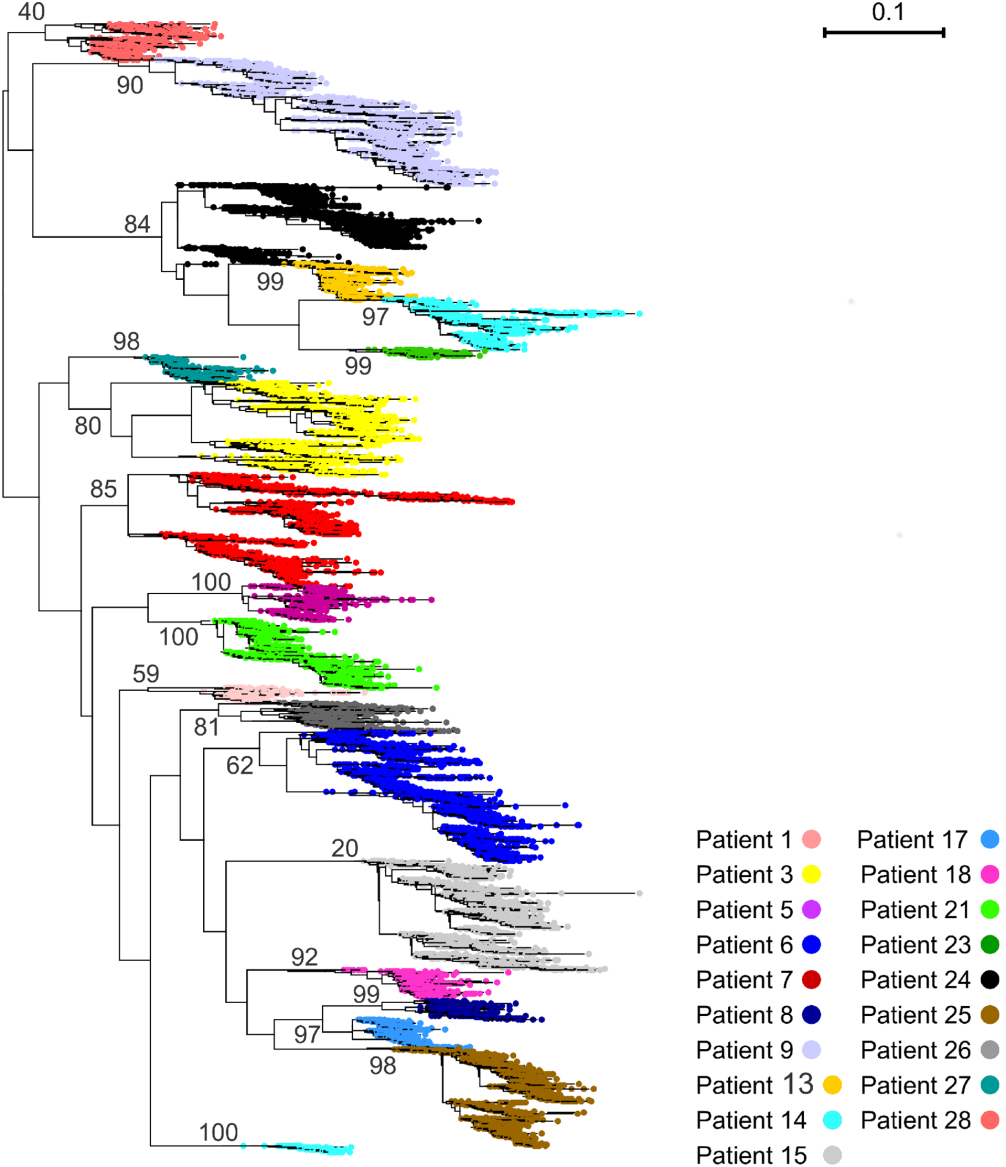
Maximum likelihood phylogenetic tree of C2-V3-C3 nucleotide sequences from patients included in the study. Patient-related clusters are identified in different colors as indicated at the bottom of the figure. The vertical size of the clusters is proportional to the number of reads in the cluster and the horizontal size of the clusters shows their maximum genetic depth. Bootstrap values are given on branches. Branch lengths are proportional to the number of nucleotide substitutions per aligned site (bar = 0.1 substitutions).

Viruses from 15 out of 19 patients were identified as HIV-1 BF intersubtype by analyzing the *env* nucleotide sequence using the NCBI genotyping tool (http://www.ncbi.nlm.nih.gov/projects/genotyping/formpage.cg0) while the remaining were ascribed to HIV-1 B subtype (Pat13, Pat23, Pat24). An individual (Pat14) showed viruses belonging to BF and B subtypes (see below). The HIV genomic characterization was further confirmed by maximum-likelihood phylogenetic analysis of the sequences studied here (Figure 1) together with HIV-1 subtype reference sequences from the Los Alamos National Laboratory (http://www.hiv.lanl.gov/content/index).

At baseline, among all 19 patients, the mean (±SD) HIV viral load was 4.89 log copies/mL (±1.38). The mean HIV viral load at baseline among the six patients from group A (those showing sustained optimal virological responses to HAART) was 4.66 log copies/mL (±0.61), and the mean CD4+ T cell count was 262 cell/µL (±80).

Among the thirteen patients from group B (those failing to HAART), the mean (±SD) plasma HIV viral load level and CD4 T cell count were 4.30 log HIV copies/mL (±1.45) and 460 cells/µL (±243), respectively when the HIV strains harbored a higher predominance of R5 tropism (82.4%±18.92). Once the antiretroviral therapy was initiated, and still exhibiting a sustained R5-tropic virus predominance, such parameters did not vary significantly (4.24±1.66 log HIV copies/mL, and 324.6±146.6 cells/µL, respectively). However, the HIV viral load (mean±SD) measured under HAART among those patients harboring X4-using variants appeared slightly lower (3.72±1.87 log HIV copies/mL; p = 0.07). At baseline, neither the CD4 T cell count nor the HIV viral load levels showed any correlation with the prevalence of HIV X4-using variants into the viral population (p>0.05 by Spearman Test).

Before antiretroviral therapy initiation, the relative abundance of X4 tropic strains (median [IQR]) between sustained optimal virological responders to HAART -group A- and patients failing to HAART –group B- showed no significant differences (2.2%[0.5–56.9] vs. 4.1% [0–17.8]), respectively; p = 0.89). At baseline, the HIV tropism prediction in patients from group A showed that 4 out of 6 individuals exhibited HIV isolates with R5 tropism predominance (98.9% [97.8–100]). On the contrary, the remaining two patients (Pat 1, and 27) were predicted to be predominantly infected with X4-using variants (75.2% and 95.6%, respectively).

During the follow-up of the thirteen patients from group B, four distinguishable dynamics of the HIV tropism were observed. First, those HIV isolates harboring a sustained tropism either R5 predominance (Pat 3, 6, 7, 13, 15, 17, 18, 21, 24), or sustained X4 tropism (patient 23); second, those viral isolates that switched from R5 to X4 tropism (Pat 14); third, HIV variants from an individual (Pat 9) that firstly appeared as X4-using and switched to R5 during the longitudinal study; and lastly, viral variants characterized from an individual (Pat 25) that experienced R5-to-X4 switch followed by a X4-to-R5 reversion. The coreceptor usage did not show any significant correlation with the HIV viral load (p = 0.55) but it should be taken cautiously because our patients were on HAART.

The agreement between geno2pheno (g2p) and HIVcoPred or, the position-specific scoring matrix (PSSM) as bioinformatics tools for genotypic interpretation of HIV-1 tropism showed an overall 92% of concordance between the first two since 3 out of 41 R5-variants were classified as X4 by HIVcoPred, and 2 out of 17 X4-using were R5 by HIVcoPred. The comparison between g2p and PSSM exhibited a concordance of 82%. Such value included a complete correlation among R5-using variants but the disagreement was found in 11 out of 19 sequences with scores associated with X4-usage that were interpreted as R5-tropic by PSSM.

### Phylogenetic inferences and evolution of HIV R5 and X4-using variants

**Figure 2:**
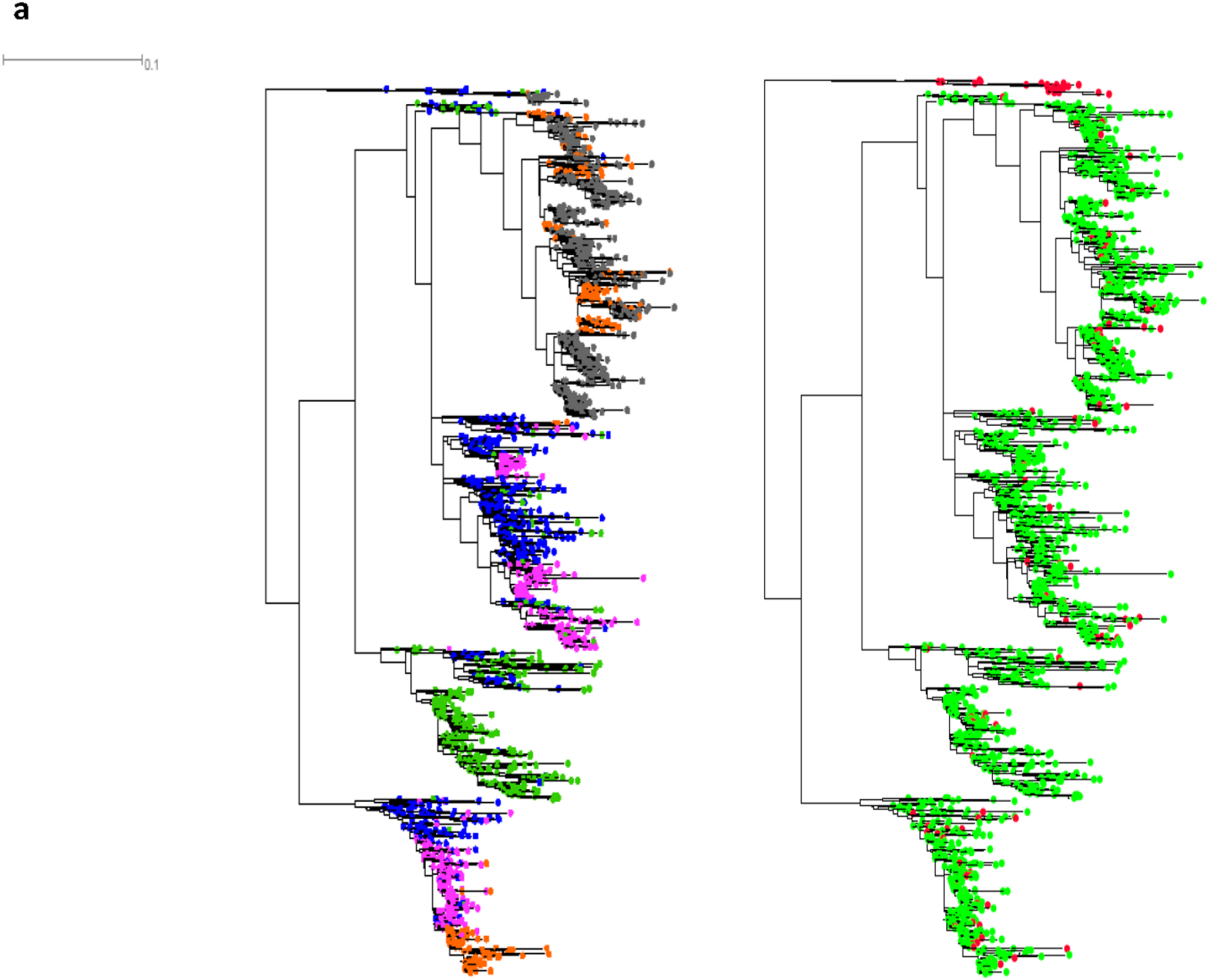
Twin maximum likelihood phylogenetic trees of C2-V3-C3 nucleotide sequences showing sampling time and tropism. Three exemplary twin trees obtained from patient 3 (Figure 2a), 7 (Figure 2b), and 25 (Figure 2c). The other trees are shown as Supporting Information. For each patient, the left tree depicts the distribution of viral variants according to the sampling time (blue: first, green: second, pink: third, orange: fourth, gray: fifth); the right tree shows the relationships among viral variants according to tropism (X4-using variants: red, R5-using variants: light green). The vertical size of the clusters is proportional to the number of reads in the cluster and the horizontal size of the clusters shows their maximum genetic depth. Branch lengths are proportional to the number of nucleotide substitutions per aligned site (bar = 0.1 substitutions).

Phylogenetic analysis allowed the recovery of strongly supported monophyletic groups of sequences corresponding to each patient, except for patients 14 and 24 (Figure 1). Sequences from each sampling point and from temporally adjacent ones displayed a tendency to cluster together in some of the patient clades (patients 3, 6, 7, 9, 13, 15, 20, 21, 24, 25 and 28; Figure 2; Figures S2-S17). However, the majority of these clades displayed a clear intermingling of sequences from different sampling times, indicating the existence of various viral lineages that persist along the infection. Furthermore, in no case sequences from a determined sampling point constituted a monophyletic group. Patient 14 presumably experienced a superinfection during the course of the follow-up (Figure S2; see below). The lack of resolution for patient 24 sequences possibly obeys to its basal position at the clade corresponding to subtype B sequences [41], [42] (Figure S14).

Given that many nodes of the single patient clades showed very low bootstrap supports, with an important proportion of tree nodes unrecovered in the bootstrapped trees (Table 2), the observed apparent complexity of viral populations was assessed using constrained analyses that were compared to unconstrained ones using approximately unbiased (AU) and non-scaled bootstrap probability (NP) tests. All the constrained trees obtained presented poor likelihoods as compared to the trees obtained in unconstrained searches, supporting the existence of several viral lineages evolving independently along the infection (Figs. 2 and S18-; Table 3).

**Table 2:** Bootstrap (*B*) and Shimodaira-Hasegawa (*SH*) supports observed inside single-patient datasets^1^.

		Patient																		
		1	3	5	6	7	8	9	13	14	15	17	18	21	23	24	25	26	27	28
	MN	16	14	13	14	17	19	15	17	16	14	16	12	15	24	16	12	20	19	16
B	MD	6	4	1	4	9	11	4	1	8	3	7	6	6	9	8	3	12	5	8
	SD	23	19	20	19	20	21	21	21	21	19	21	20	18	26	23	17	21	23	20
	MN	0.46	0.62	0.48	0.56	56.9	0.53	0.56	0.53	0.55	0.55	0.52	0.54	0.55	0.63	0.52	0.51	0.66	0.51	0.61
SH	MD	0.46	0.76	0.45	0.74	73.9	0.76	0.73	0.63	0.66	0.68	0.55	0.62	0.72	0.76	0.71	0.53	0.77	0.46	0.76
	SD	0.34	0.32	0.32	0.34	0.33	0.31	0.33	0.35	0.33	0.32	0.38	0.34	0.34	0.31	0.27	0.34	0.27	0.36	0.31

1 Mean (*MN*), median (*MD*), standard deviation (*SD*).

**Table 3:** Results of approximately unbiased (AU) and non-scaled bootstrap probability (NP) tests^1^.

		Patient												
		1	3	6	7	9	13	15	17	20	21	24	25	29
AU	U^2^	0.823	0.999	0.999	0.537	0.953	0.997	0.999	0.730	0.999	0.999	0.999	0.579	0.999
	FV^3^	0.177	-	-	0.463	0.048	-	-	-	-	-	-	0.447	-
	LE^4^	NA^5^	**5e-04**	**2e-06**	**3e-32**	**8e-06**	**0.003**	**1e-05**	0.270	**5e-85**	**5e-07**	**8e-47**	**0.005**	**8e-43**
NP	U	0.817	0.999	0.999	0.526	0.948	0.997	0.999	0.716	0.999	0.999	0.999	0.575	0.999
	FV	0.183	-	-	0.474	0.052	-	-	-	-	-	-	0.422	-
	LE	NA	**4e-06**	**2e-06**	**4e-14**	**2e-05**	**0.003**	**1e-05**	0.284	**5e-23**	**1e-05**	**3e-16**	**0.002**	**8e-37**

1 Significant differences (*p*<0.01) in bold.

2 Unconstrained.

3 Fitness Valley.

4 Lineage extinction.

5 Not applicable.

Phylogenetic trees also indicated a complex scenario for the emergence of X4 usage. Several individual patient topologies showed that X4 variants can evolve in multiple, independent events along the infection (Figure 2; Figures S1–S16). However, some patient clades displayed subclades composed mostly of X4 sequences, supporting the hypothesis that the R5 to X4 transition can, in some cases, imply a transition through a fitness valley, where intermediate variants exhibited a reduced replication capacity and were present at much lower frequency (Patients 1, 7, 9 and 25; Figs. 2 and S2–S18; Table 3). Thus, our results are compatible with data published previously indicating that CXCR4 usage can emerge in multiple lineages, and that the occurrence of fitness valleys depends on viral genetic background [36].

**Figure 3:**
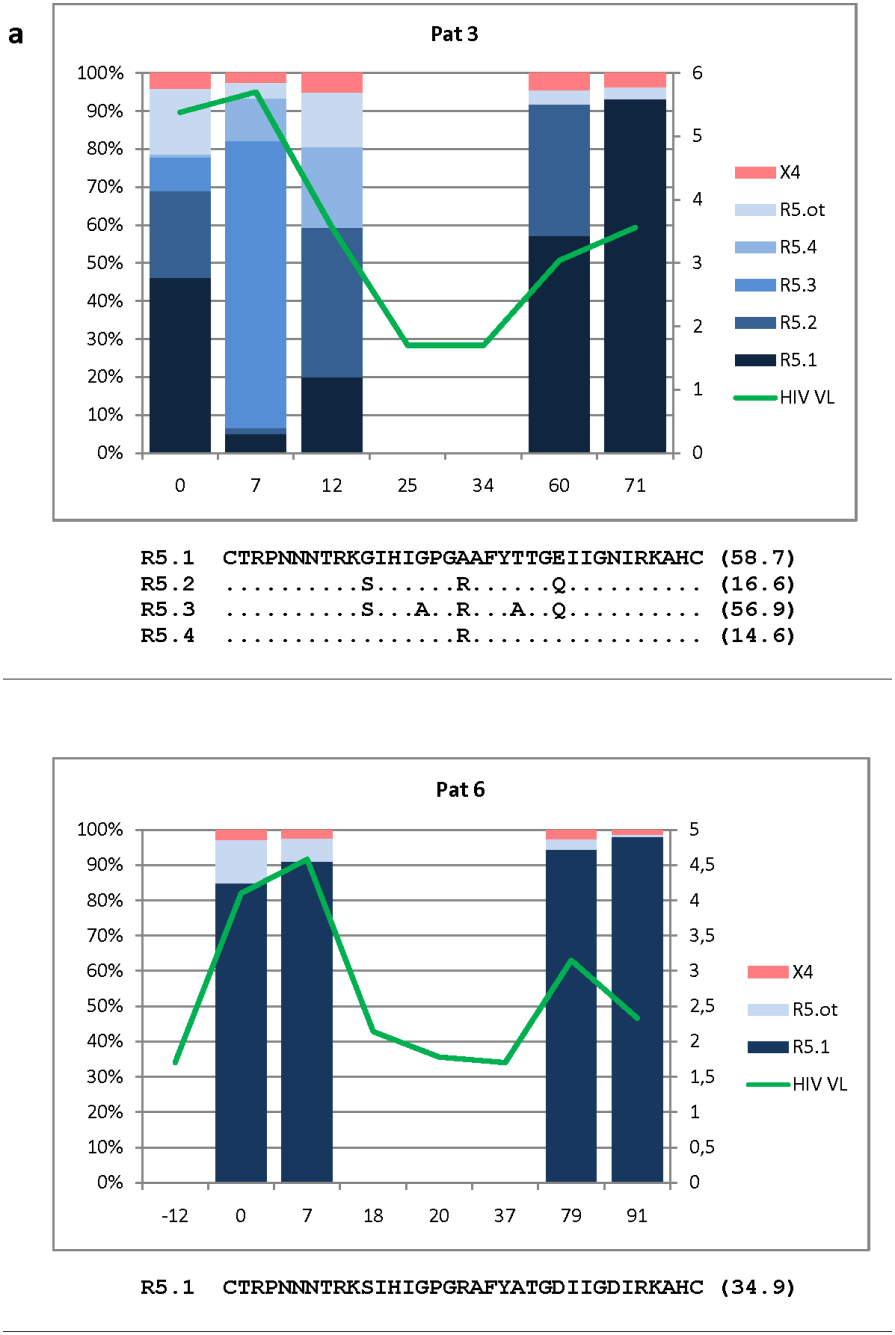
Intra-patient relative abundance of R5 and X4-using variants identified over time. Longitudinal analysis of the relative abundance (y left axis: percentage) of R5 (blue range) and X4 (red range) variants at different time points (x axis: sampling times in months). At the first sampling time, the most abundant variant is identified by darker color and named as “R5.1” or “X4.1”, accordingly. Colors become clearer according to the decrease in their relative abundance. “R5.ot” and “X4.ot” include those viral variants with very low relative abundance. For subsequent sampling times, the color palette is respected following the first one in order to allow correlating the longitudinal variation of the viral variants first identified. HIV viral load are represented (y right axis; as log copies/ml). The V3 amino acid sequence (and between brackets, their relative false positive rate by geno2pheno) of the most common variants in each patient is shown.

The dynamics of HIV evolution according to the coreceptor usage appeared also to be host-related. Among those that were analyzed longitudinally, the X4-using variants exhibited alternative behaviors after their emergence. Several subjects (3, 6, 7, 15, 17, 18, and 25) exhibited dispersed X4-variants that were phylogenetically intermingled among R5-using ones. The vast majority did not reach a substantial frequency in the viral quasispecies population, hence allowing presuming a neutral evolution or alternatively, the existence of negative selection pressure. In contrast, other emerging X4-using variants did not go extinction, suggesting the existence of positive selection processes (Pat 3, 7, and 25; Figure 2) that conducted to the establishment of X4-subclade. Some individuals showed particularly interesting cases. For instance, the R5-to-X4 switch in subject 25 occurred at thirty-one months from baseline but then, after nine months, a reversion to R5-using variants predominance was observed. Remarkably, the R5-using variants that predominated at 40 months were closely related to sequences sampled at times 0 and 8 months, indicating that the reversion to R5 usage responded to the rise of lineages that were already present at the beginning of the follow-up (Fig. 2c; Fig. 3d). Patient 9 also verified a coreceptor switch but it was from X4-to-R5 switch. In this host, two X4 haplotypes were indentified; one of them was present at the beginning of the follow-up and could have become extinct after the second sampling time. The R5 lineages present at 84 and 95 months of the follow-up emerged independently of this lineage (Fig. S6; Fig. 3b). These observations reinforce the idea of the existence of complex lineage assemblages evolving along the HIV infection.

Another case of R5-to-X4 switch was observed in subject 14 (Fig. S8; Fig. 3e). However, taking into account that the X4-variants observed were both phylogenetically distant and temporally separated from the unique pre-existent R5-haplotype, such apparent reversion in the HIV tropism could be probably explained by two independent events of infection. At the fourth and fifth sampling times, when the HIV viral load was fluctuating, two X4-using haplotypes emerged and became highly predominant accompanied by R5-using variants not related with that found previously at baseline.

### Intrapatient dynamics of the HIV quasispecies evolution and the coreceptor usage

Here, at least in a single sampling time during the follow-up, many patients showed the coexistence of both R5 and X4-using variants with dissimilar frequencies each. Under drug naïve conditions, among the 13 patients able to study prior to antiretroviral therapy initiation, X4-using variants were detected with an intra-patient prevalence ranging from 0% to 95.6% (median [IQR]: 4.1% [2.1%–37.5%]). Similarly, the prevalence of intra-patient X4- using variants among drug-experienced patients ranged from 0% to 88.2% (4.1% [1.6%–27.0%].

The intra-host analysis of V3 amino acid sequence dynamics showed that the alternative coreceptor R5 or X4 usage may imply one (i.e. Pat 9) or several (i.e. Pat 7) amino acid replacements. The intra-patient quasispecies diversity and variability (by Shannon entropy) at a protein level ranged from 0.016 subs/site to 0.264 subs/site and from 0.21 to 0.62, respectively assessing different selection pressures over time. Both parameters showed no correlation with intra-host prevalence of X4-using variants (p>0.05, by Spearman test). Likewise, no significant differences (Mann-Whitney test p>0.05) were observed when comparing the median [IQR] diversity and variability of amino acid sequences between those sampling times showing X4 variants predominance (0.032 [0.023–0.056] and 0.27 [0.24–0.38], respectively), against those exhibiting R5 variants predominance (0.049 [0.030–0.091] and 0.36 [0.29–0.42], respectively).

In this scenario, the next step of this study was to analyze longitudinally at intra-tropism level, the dynamics of the HIV quasispecies composition. Among our patients, two models were observed (Figure 3; S18-S30). On the one hand, HIV haplotypes from Pat 6, 13 and 15 showed null or, small variations in their relative frequencies of R5 haplotypes during the study period (75.8±10.5 months), despite HIV replication fluctuations (Fig. 3a, 3d, 3e, S20, S23, S24). In contrast, other patients showed larger variations in their HIV quasispecies composition during a similar period of time (79.6±23.2 months), depicting pronounced fluctuations of haplotype frequencies (Pat 3, 7, 9, 14, 21, 24, 25) (Figure 3, S19, S21, S22, S27, S28, S29).

As expected, this second group exhibited significant higher values than the former on its quasispecies heterogeneity-related parameters (diversity and variability) (p<0.001 and p<0.0016 respectively). Nevertheless, the period of time involved in the follow-up was similar but considering that the interval between sampling times was irregular, changes into quasispecies composition could not be advised properly and further research is deserved.

### Sequence Analysis by Two Sample Logo (TSL)

**Figure 4:**
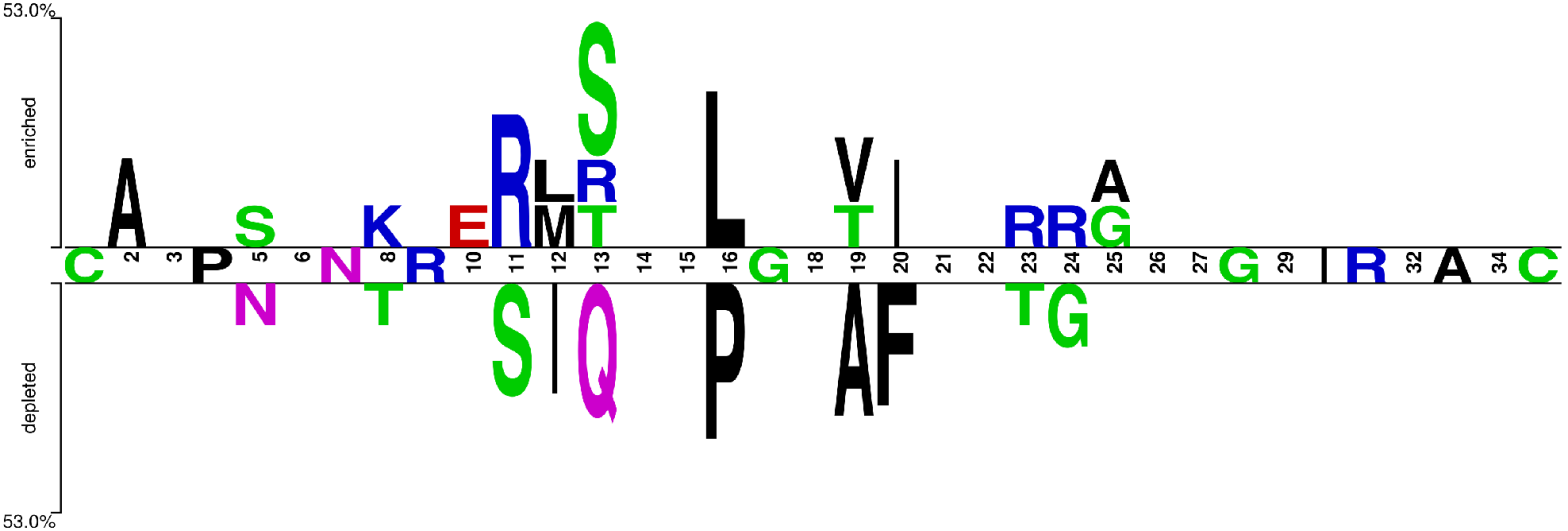
Comparative frequency of amino acid residues between R5- and X4-viruses. By using the two sample logo (TSL) amino acid residues with significant difference in the frequency between the two datasets (R5-tropic n = 41, lower panel; X4-tropic n = 19, upper panel) are shown at the specific sites in the HIV gp-120 V3 sequences. Residues between the two panels denoted those with the equal frequency in two datasets; when such frequency was approximately equal, positions showed no residues.

The analysis of the relative frequencies of amino acids at a position in the X4- and R5-tropic datasets aligned sequences showed significant differences. It was found that positively charged amino acids such as Lys (position 8) and Arg (positions 11, 13, 23, 24) and the large ones such as Ile (position 12) and Phe (position 20) are present at higher frequencies in the X4-tropic, while the frequency of occurrence of Asn (position 5) and small amino acids (Gly, Ala, Ser, Pro, etc.) was relatively higher in the R5-tropic sequences (Figure 4).

## Discussion

Human immunodeficiency virus type 1 (HIV-1) tropism for the chemokine receptors CCR5 and CXCR4 was shown to be associated with disease progression. HIV-R5 viruses are generally predominant at early stages of HIV-1 infection, whereas the emergence of X4-viruses generally occurs at later stages. However, the evolution of virus tropism under HAART is controversial and the selective mechanisms driving emergence of CXCR4-using variants are still unrevealed [43], [44].

In the present study we applied a phylogenetic framework to a large dataset generated by ultra deep pyrosequencing (UDPS) aimed at studying the population dynamics of HIV coreceptor usage. To achieve this aim, a longitudinal follow-up was carried out for up to 11 years of HIV infected patients most of whom were HAART-naïve at baseline while others were experienced ones.

In our study, in the majority (77%) of HAART-naive patients, R5-using viruses were detected. Taking into account that X4 virus is more likely to be detected in more advanced disease, its presence at baseline could be explained considering that late diagnosis of HIV infection occurs in a substantial proportion of patients newly diagnosed as HIV-positive in Argentina [45] and other countries [46]. The presence of both R5- and X4-tropism in naïve and long term treated patients at advanced stage of infection argues against a role of HAART itself in the tropism switch. Instead, the infection progress to later stages with eventual immunological impairment might propitiate an increasing appearance of X4-tropic viruses. In contrast with previous assumptions but in line with other reports [47], [48], we found that the median CD4 cell count was not significantly associated with X4-coreceptor usage virus. This inconsistency could be explained considering the plausible late stage of infection among our patients allowing the R5 viruses develop mutations that increase their cytophatic capacity on CD4 T cells.

After establishing inter- and intra-host phylogenetic relationships among HIV-1 isolates characterized during this longitudinal study, the persistence of several viral lineages evolving independently along the infection was statistically supported, indicating a complex scenario for the evolution of viral quasispecies. In agreement with recent studies, some viral populations displayed significant evidence of stochastic tunneling processes [36], [37], which supports the evolution of X4 variants through fitness valleys, whereas viruses from other patients displayed no evidence of this phenomenon. Considering that HIV, like other RNA viruses, can evolve rapidly over short periods of time, the switch in the coreceptor usage could also occur very rapidly as well. As a consequence, the selection process on viral variants is difficult to prove. However, the time needed to switch from R5- to X4 has been estimated around 4 years post-infection [37]. Here, as it is already known, HIV variants using either CXCR4 or CCR5 were found to coexist in plasma.

Furthermore, our data showed that X4 usage can evolve in multiple, independent events along the infection. The specific mutational pathway that led to CXCR4-usage was different from viruses found in each individual, and is likely to be at least partially constrained by the viral background. The constraints on the R5 to X4 mutational pathways and the low fitness of transitional intermediate variants support that X4 variants were present at extremely low frequencies in some patients, in agreement with the results reported recently [49]. In this context, some individuals exhibited X4-using viral variants that were able to overcome such fitness valley and became predominant. Phylogenetically they appeared as intra-host monophyletic groups or clades dominated largely by X4 sequences. Alternatively, other X4-using variants seem to belong to lineages that failed to overcome such fitness valley and thus appeared intermingled with R5-variants along the phylogenetic trees and were present at low frequencies only detectable by using deep-sequencing techniques. Apparently, such alternative pathways are not mutually exclusive, as the phylogenies that displayed X4-clades also presented spare, interspersed X4 sequences that might correspond to evolutionary dead ends.

Interestingly, a reversion in the predominance from X4-to-R5 using variants was also observed. This reversion was coincident with a strong treatment-induced suppression of HIV viral load and reduced T-cell turnover, as reported previously [50]. Hence, the dynamics of HIV evolution of coreceptor usage appeared to depend on the individual studied as well as the genotype of the variant transmitted. In this sense, among those infected predominantly with R5-tropic viruses we observed two dynamics on its haplotypes composition. On the one hand, those showing little variations during the longitudinal study suggesting that proliferation of latently infected cells could be the main mechanism accounting for virus persistence. On the other hand, those exhibiting more dramatic changes in their quasispecies could reflect residual virus replication despite HAART [44].

The intra-host X4-predominance did not correlate with parameters of quasispecies heterogeneity during this longitudinal study. Instead, a positive correlation was reported by two cross-sectional studies [21], [51]. The different study design is a key explanation to such discrepancy. Besides, neither the mutational pathways toward X4-using variants necessarily implied heterogeneity accumulation, nor the X4-variants appeared intrinsically more heterogeneous. When comparing the V3-loop amino acid sequences, a unique discrepancy was observed among some variants ascribed to R5 or X4-inferred tropism. But these changes followed a pattern that revealed that positively charged and larger amino acids are more frequent in X4- than in R5-tropic sequences, as reported recently [25].

In our study, the HIV-1 tropism assessment did not include phenotypic assays as gold standard. This limitation in conjunction with both the unavailability of peripheral blood mononuclear cells [52] and the predominance of HIV-1 BF intersubtype isolates [53] may impair the X4-using variants detection. We used three alternative methods for tropism determination based on the genotypic prediction of HIV-1 coreceptor usage through bioinformatics tools based on V3 loop viral sequences. Although we found a good correlation between them, as reported previously [54], the use of ultra-deep sequencing techniques restricted the tropism analysis to the V3 loop without taking into consideration other changes in the viral envelope that may influence coreceptor usage and viral fitness. Particularly, substitutions in the V1, V2, C4 regions of gp120 and gp41 protein as well as differences in Env glycosylation patterns are known to play an important role in the determination of the coreceptor usage and may compensate loss-of-fitness mutations in V3 [49], [55], [56], [57], [58], [59], [60].

In conclusion, the dynamics of HIV coreceptor usage showed several viral lineages evolving independently along the infection accompanied by a complex evolution of viral quasispecies. Several HIV R5 and/or X4 haplotypes were characterized among the patients studied, exhibiting a dissimilar inter- and intra-patient predominance as a component of the viral population still on antiretroviral therapy. Furthermore, our analyses suggest that, besides becoming dominant after population-level switches, minor proportions of X4 viruses might exist along the infection, perhaps even at early stages of it. The fate of these minor variants might depend on both viral and host factors.

## Supporting Information

Figure S1 **Phylogenetic trees before (A) and after (B) removal of outlier sequences (indicated by red diamonds in panel A).** The tree corresponds to patient 3 sequences. The numbers of outliers and the disposition of the corresponding terminals were equivalent for the rest of patients (not shown).
(TIF)

Figure S2 **Twin trees obtained from patient 1.** The left tree depicts the distribution of viral variants according to the sampling time (blue: first, green: second, pink: third, orange: fourth, gray: fifth); the right tree shows relationships among viral variants according to tropism (X4-using variants: red, R5-using variants: light green). The vertical size of the clusters is proportional to the number of reads in the cluster and the horizontal size of the clusters shows their maximum genetic depth. Branch lengths are proportional to the number of nucleotide substitutions per aligned site (bar = 0.1 substitutions).
(TIF)

Figure S3 **Twin trees obtained from patient 5.** The left tree depicts the distribution of viral variants according to the sampling time (blue: first, green: second, pink: third, orange: fourth, gray: fifth); the right tree shows relationships among viral variants according to tropism (X4-using variants: red, R5-using variants: light green). The vertical size of the clusters is proportional to the number of reads in the cluster and the horizontal size of the clusters shows their maximum genetic depth. Branch lengths are proportional to the number of nucleotide substitutions per aligned site (bar = 0.1 substitutions).
(TIF)

Figure S4 **Twin trees obtained from patient 6.** The left tree depicts the distribution of viral variants according to the sampling time (blue: first, green: second, pink: third, orange: fourth, gray: fifth); the right tree shows relationships among viral variants according to tropism (X4-using variants: red, R5-using variants: light green). The vertical size of the clusters is proportional to the number of reads in the cluster and the horizontal size of the clusters shows their maximum genetic depth. Branch lengths are proportional to the number of nucleotide substitutions per aligned site (bar = 0.1 substitutions).
(TIF)

Figure S5 **Twin trees obtained from patient 8.** The left tree depicts the distribution of viral variants according to the sampling time (blue: first, green: second, pink: third, orange: fourth, gray: fifth); the right tree shows relationships among viral variants according to tropism (X4-using variants: red, R5-using variants: light green). The vertical size of the clusters is proportional to the number of reads in the cluster and the horizontal size of the clusters shows their maximum genetic depth. Branch lengths are proportional to the number of nucleotide substitutions per aligned site (bar = 0.1 substitutions).
(TIF)

Figure S6 **Twin trees obtained from patient 9.** The left tree depicts the distribution of viral variants according to the sampling time (blue: first, green: second, pink: third, orange: fourth, gray: fifth); the right tree shows relationships among viral variants according to tropism (X4-using variants: red, R5-using variants: light green). The vertical size of the clusters is proportional to the number of reads in the cluster and the horizontal size of the clusters shows their maximum genetic depth. Branch lengths are proportional to the number of nucleotide substitutions per aligned site (bar = 0.1 substitutions).
(TIF)

Figure S7 **Twin trees obtained from patient 13.** The left tree depicts the distribution of viral variants according to the sampling time (blue: first, green: second, pink: third, orange: fourth, gray: fifth); the right tree shows relationships among viral variants according to tropism (X4-using variants: red, R5-using variants: light green). The vertical size of the clusters is proportional to the number of reads in the cluster and the horizontal size of the clusters shows their maximum genetic depth. Branch lengths are proportional to the number of nucleotide substitutions per aligned site (bar = 0.1 substitutions).
(TIF)

Figure S8 **Twin trees obtained from patient 14.** The left tree depicts the distribution of viral variants according to the sampling time (blue: first, green: second, pink: third, orange: fourth, gray: fifth); the right tree shows relationships among viral variants according to tropism (X4-using variants: red, R5-using variants: light green). The vertical size of the clusters is proportional to the number of reads in the cluster and the horizontal size of the clusters shows their maximum genetic depth. Branch lengths are proportional to the number of nucleotide substitutions per aligned site (bar = 0.1 substitutions).
(TIF)

Figure S9 **Twin trees obtained from patient 15.** The left tree depicts the distribution of viral variants according to the sampling time (blue: first, green: second, pink: third, orange: fourth, gray: fifth); the right tree shows relationships among viral variants according to tropism (X4-using variants: red, R5-using variants: light green). The vertical size of the clusters is proportional to the number of reads in the cluster and the horizontal size of the clusters shows their maximum genetic depth. Branch lengths are proportional to the number of nucleotide substitutions per aligned site (bar = 0.1 substitutions).
(TIF)

Figure S10 **Twin trees obtained from patient 17.** The left tree depicts the distribution of viral variants according to the sampling time (blue: first, green: second, pink: third, orange: fourth, gray: fifth); the right tree shows relationships among viral variants according to tropism (X4-using variants: red, R5-using variants: light green). The vertical size of the clusters is proportional to the number of reads in the cluster and the horizontal size of the clusters shows their maximum genetic depth. Branch lengths are proportional to the number of nucleotide substitutions per aligned site (bar = 0.1 substitutions).
(TIF)

Figure S11 **Twin trees obtained from patient 18.** The left tree depicts the distribution of viral variants according to the sampling time (blue: first, green: second, pink: third, orange: fourth, gray: fifth); the right tree shows relationships among viral variants according to tropism (X4-using variants: red, R5-using variants: light green). The vertical size of the clusters is proportional to the number of reads in the cluster and the horizontal size of the clusters shows their maximum genetic depth. Branch lengths are proportional to the number of nucleotide substitutions per aligned site (bar = 0.1 substitutions).
(TIF)

Figure S12 **Twin trees obtained from patient 21.** The left tree depicts the distribution of viral variants according to the sampling time (blue: first, green: second, pink: third, orange: fourth, gray: fifth); the right tree shows relationships among viral variants according to tropism (X4-using variants: red, R5-using variants: light green). The vertical size of the clusters is proportional to the number of reads in the cluster and the horizontal size of the clusters shows their maximum genetic depth. Branch lengths are proportional to the number of nucleotide substitutions per aligned site (bar = 0.1 substitutions).
(TIF)

Figure S13 **Twin trees obtained from patient 23.** The left tree depicts the distribution of viral variants according to the sampling time (blue: first, green: second, pink: third, orange: fourth, gray: fifth); the right tree shows relationships among viral variants according to tropism (X4-using variants: red, R5-using variants: light green). The vertical size of the clusters is proportional to the number of reads in the cluster and the horizontal size of the clusters shows their maximum genetic depth. Branch lengths are proportional to the number of nucleotide substitutions per aligned site (bar = 0.1 substitutions).
(TIF)

Figure S14 **Twin trees obtained from patient 24.** The left tree depicts the distribution of viral variants according to the sampling time (blue: first, green: second, pink: third, orange: fourth, gray: fifth); the right tree shows relationships among viral variants according to tropism (X4-using variants: red, R5-using variants: light green). The vertical size of the clusters is proportional to the number of reads in the cluster and the horizontal size of the clusters shows their maximum genetic depth. Branch lengths are proportional to the number of nucleotide substitutions per aligned site (bar = 0.1 substitutions).
(TIF)

Figure S15 **Twin trees obtained from patient 26.** The left tree depicts the distribution of viral variants according to the sampling time (blue: first, green: second, pink: third, orange: fourth, gray: fifth); the right tree shows relationships among viral variants according to tropism (X4-using variants: red, R5-using variants: light green). The vertical size of the clusters is proportional to the number of reads in the cluster and the horizontal size of the clusters shows their maximum genetic depth. Branch lengths are proportional to the number of nucleotide substitutions per aligned site (bar = 0.1 substitutions).
(TIF)

Figure S16 **Twin trees obtained from patient 27.** The left tree depicts the distribution of viral variants according to the sampling time (blue: first, green: second, pink: third, orange: fourth, gray: fifth); the right tree shows relationships among viral variants according to tropism (X4-using variants: red, R5-using variants: light green). The vertical size of the clusters is proportional to the number of reads in the cluster and the horizontal size of the clusters shows their maximum genetic depth. Branch lengths are proportional to the number of nucleotide substitutions per aligned site (bar = 0.1 substitutions).
(TIF)

Figure S17 **Twin trees obtained from patient 28.** The left tree depicts the distribution of viral variants according to the sampling time (blue: first, green: second, pink: third, orange: fourth, gray: fifth); the right tree shows relationships among viral variants according to tropism (X4-using variants: red, R5-using variants: light green). The vertical size of the clusters is proportional to the number of reads in the cluster and the horizontal size of the clusters shows their maximum genetic depth. Branch lengths are proportional to the number of nucleotide substitutions per aligned site (bar = 0.1 substitutions).
(TIF)

Figure S18 **Phylogenetic analysis of Patient 1 Kec haplotypes.** The trees in left and right panels are twin trees on which isolation time and tropism, respectively, were mapped. Color codes are as in Figure 2.
(TIF)

Figure S19 **Phylogenetic analysis of Patient 3 Kec haplotypes.** The trees in left and right panels are twin trees on which isolation time and tropism, respectively, were mapped. Color codes are as in Figure 2.
(TIF)

Figure S20 **Phylogenetic analysis of Patient 6 Kec haplotypes.** The trees in left and right panels are twin trees on which isolation time and tropism, respectively, were mapped. Color codes are as in Figure 2.
(TIF)

Figure S21 **Phylogenetic analysis of Patient 7 Kec haplotypes.** The trees in left and right panels are twin trees on which isolation time and tropism, respectively, were mapped. Color codes are as in Figure 2.
(TIF)

Figure S22 **Phylogenetic analysis of Patient 9 Kec haplotypes.** The trees in left and right panels are twin trees on which isolation time and tropism, respectively, were mapped. Color codes are as in Figure 2.
(TIF)

Figure S23 **Phylogenetic analysis of Patient 13 Kec haplotypes.** The trees in left and right panels are twin trees on which isolation time and tropism, respectively, were mapped. Color codes are as in Figure 2.
(TIF)

Figure S24 **Phylogenetic analysis of Patient 15 Kec haplotypes.** The trees in left and right panels are twin trees on which isolation time and tropism, respectively, were mapped. Color codes are as in Figure 2.
(TIF)

Figure S25 **Phylogenetic analysis of Patient 17 Kec haplotypes.** The trees in left and right panels are twin trees on which isolation time and tropism, respectively, were mapped. Color codes are as in Figure 2.
(TIF)

Figure S26 **Phylogenetic analysis of Patient 20 Kec haplotypes.** The trees in left and right panels are twin trees on which isolation time and tropism, respectively, were mapped. Color codes are as in Figure 2.
(TIF)

Figure S27 **Phylogenetic analysis of Patient 21 Kec haplotypes.** The trees in left and right panels are twin trees on which isolation time and tropism, respectively, were mapped. Color codes are as in Figure 2.
(TIF)

Figure S28 **Phylogenetic analysis of Patient 24 Kec haplotypes.** The trees in left and right panels are twin trees on which isolation time and tropism, respectively, were mapped. Color codes are as in Figure 2.
(TIF)

Figure S29 **Phylogenetic analysis of Patient 25 Kec haplotypes.** The trees in left and right panels are twin trees on which isolation time and tropism, respectively, were mapped. Color codes are as in Figure 2.
(TIF)

Figure S30 **Phylogenetic analysis of Patient 28 Kec haplotypes.** The trees in left and right panels are twin trees on which isolation time and tropism, respectively, were mapped. Color codes are as in Figure 2.
(TIF)

Table S1 **UDPS results validation by a comparative analysis using conventional cloning.**
(PDF)

Table S2 **Laboratory results obtained during the follow-up (expressed as months):predicted HIV tropism (by Geno2pheno); HIV plasma viral load (log copies/mL); CD4 T-cell count (cell/mm3).**
(PDF)
