# Supplementary figures and images for: HIV-1 Tropism Dynamics and Phylogenetic Analysis from Longitudinal Ultra-Deep Sequencing Data of CCR5- and CXCR4-Using Variants
Source: PLoS One. 2014 Jul 17;9(7):e102857. doi: 10.1371/journal.pone.0102857 (PMC4102574; doi:10.1371/journal.pone.0102857)

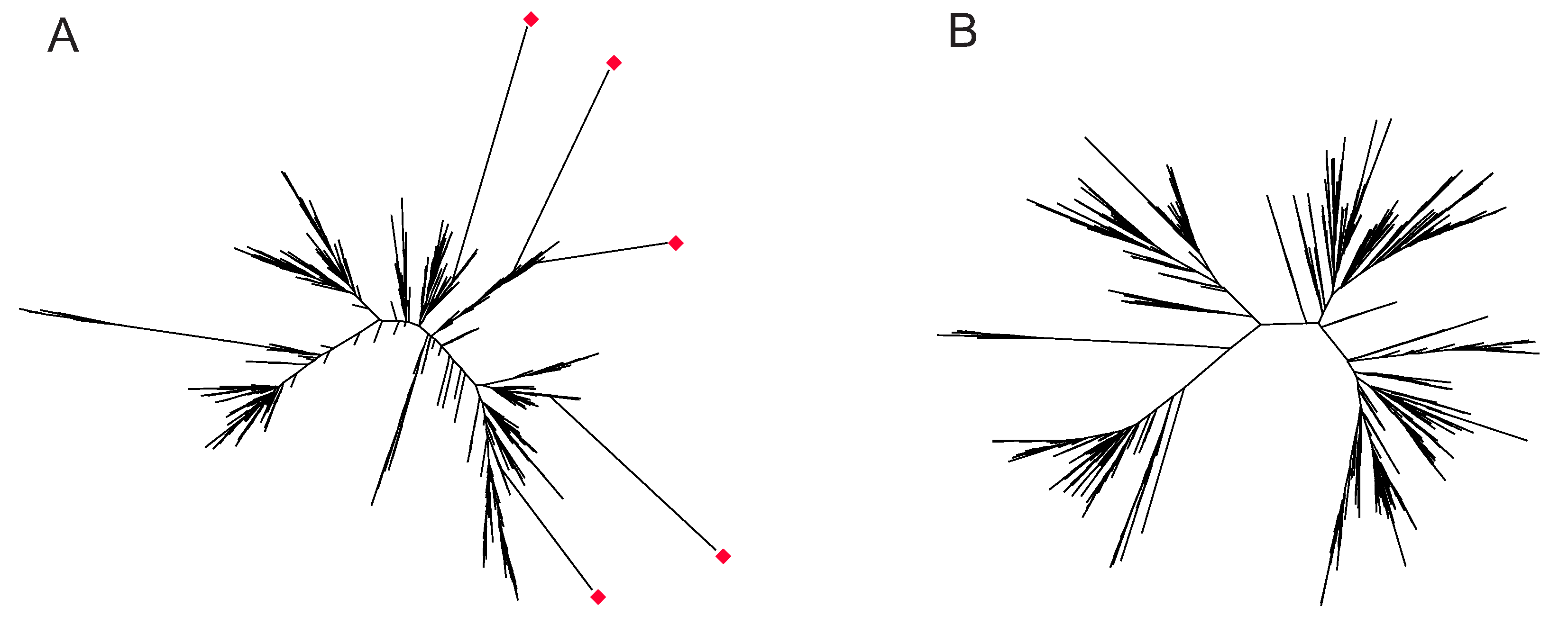

Supplement: Figure S1 — Phylogenetic trees before (A) and after (B) removal of outlier sequences (indicated by red diamonds in panel A). The tree corresponds to patient 3 sequences. The numbers of outliers and the disposition of the corresponding terminals were equivalent for the rest of patients (not shown). (TIF) [file pone.0102857.s001.tif]

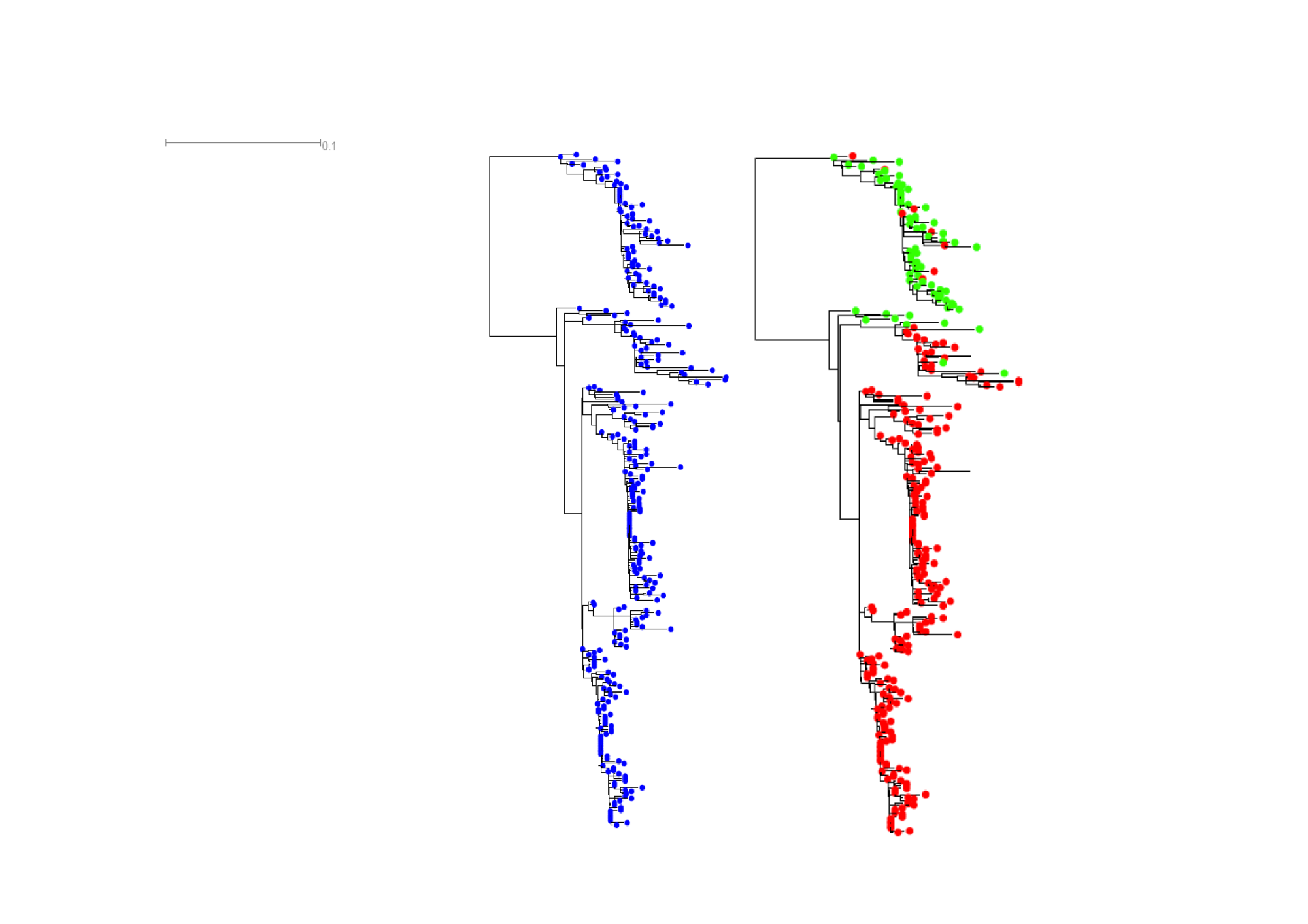

Supplement: Figure S2 — Twin trees obtained from patient 1. The left tree depicts the distribution of viral variants according to the sampling time (blue: first, green: second, pink: third, orange: fourth, gray: fifth); the right tree shows relationships among viral variants according to tropism (X4-using variants: red, R5-using variants: light green). The vertical size of the clusters is proportional to the number of reads in the cluster and the horizontal size of the clusters shows their maximum genetic depth. Branch lengths are proportional to the number of nucleotide substitutions per aligned site (bar = 0.1 substitutions). (TIF) [file pone.0102857.s002.tif]

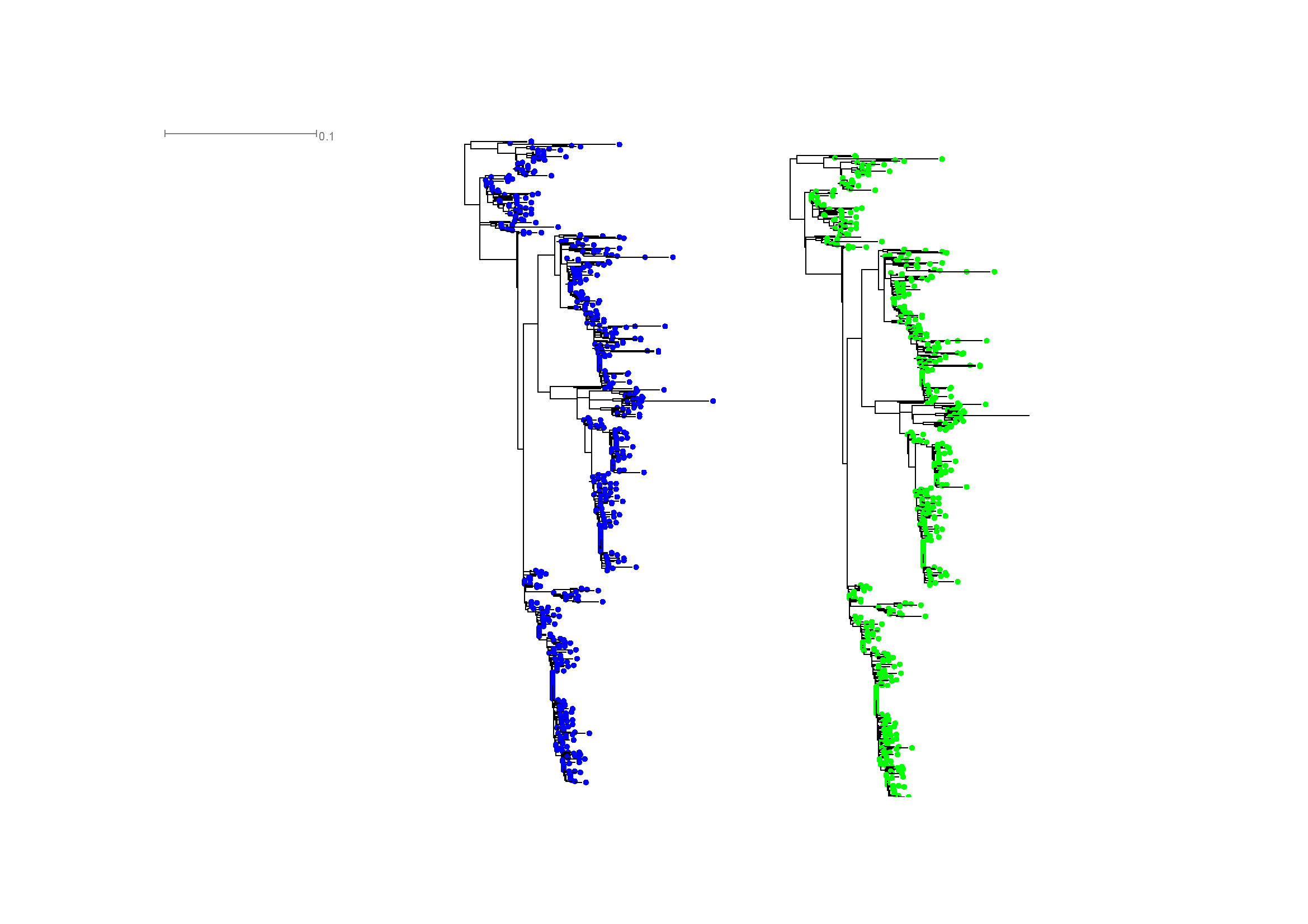

Supplement: Figure S3 — Twin trees obtained from patient 5. The left tree depicts the distribution of viral variants according to the sampling time (blue: first, green: second, pink: third, orange: fourth, gray: fifth); the right tree shows relationships among viral variants according to tropism (X4-using variants: red, R5-using variants: light green). The vertical size of the clusters is proportional to the number of reads in the cluster and the horizontal size of the clusters shows their maximum genetic depth. Branch lengths are proportional to the number of nucleotide substitutions per aligned site (bar = 0.1 substitutions). (TIF) [file pone.0102857.s003.tif]

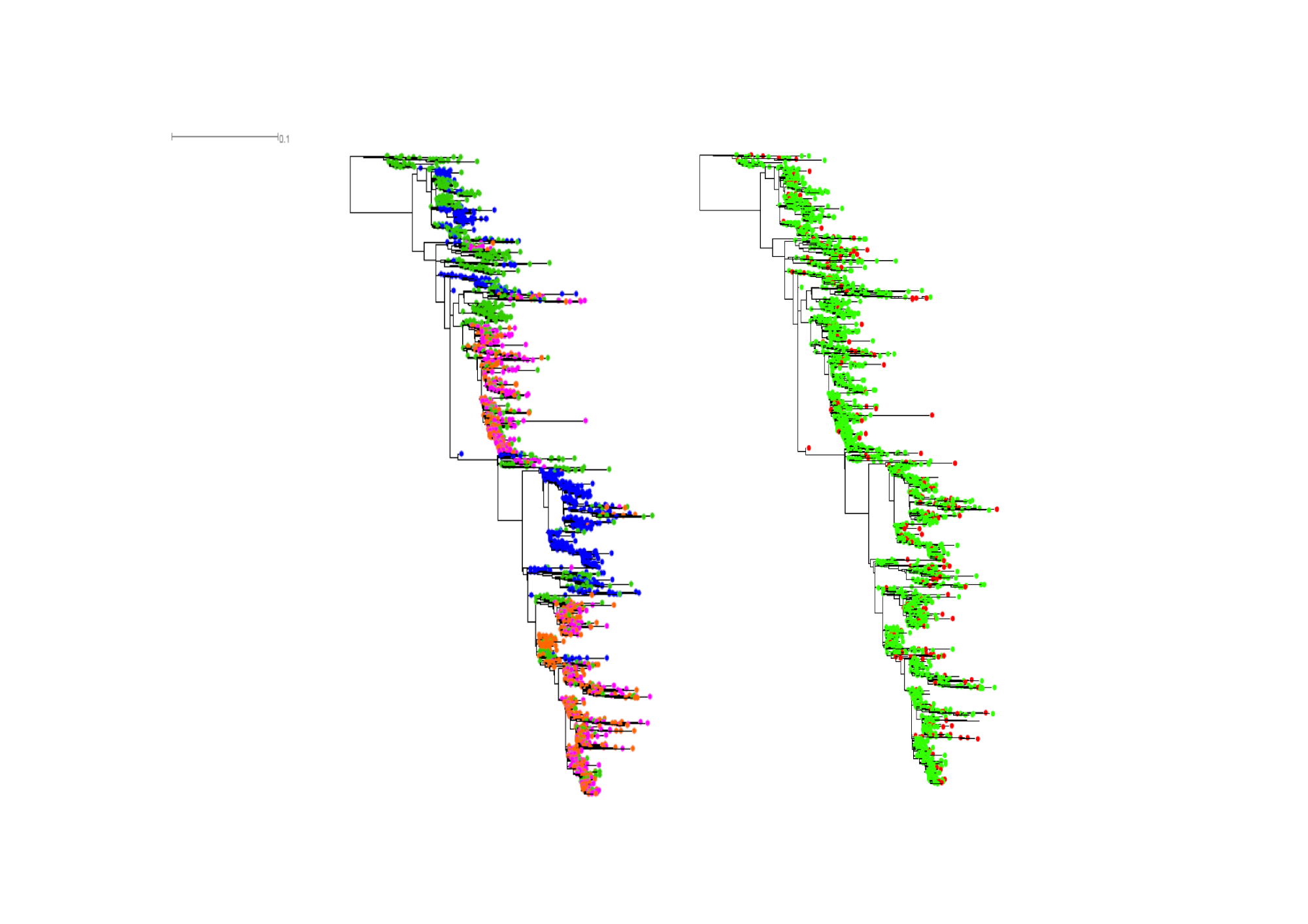

Supplement: Figure S4 — Twin trees obtained from patient 6. The left tree depicts the distribution of viral variants according to the sampling time (blue: first, green: second, pink: third, orange: fourth, gray: fifth); the right tree shows relationships among viral variants according to tropism (X4-using variants: red, R5-using variants: light green). The vertical size of the clusters is proportional to the number of reads in the cluster and the horizontal size of the clusters shows their maximum genetic depth. Branch lengths are proportional to the number of nucleotide substitutions per aligned site (bar = 0.1 substitutions). (TIF) [file pone.0102857.s004.tif]

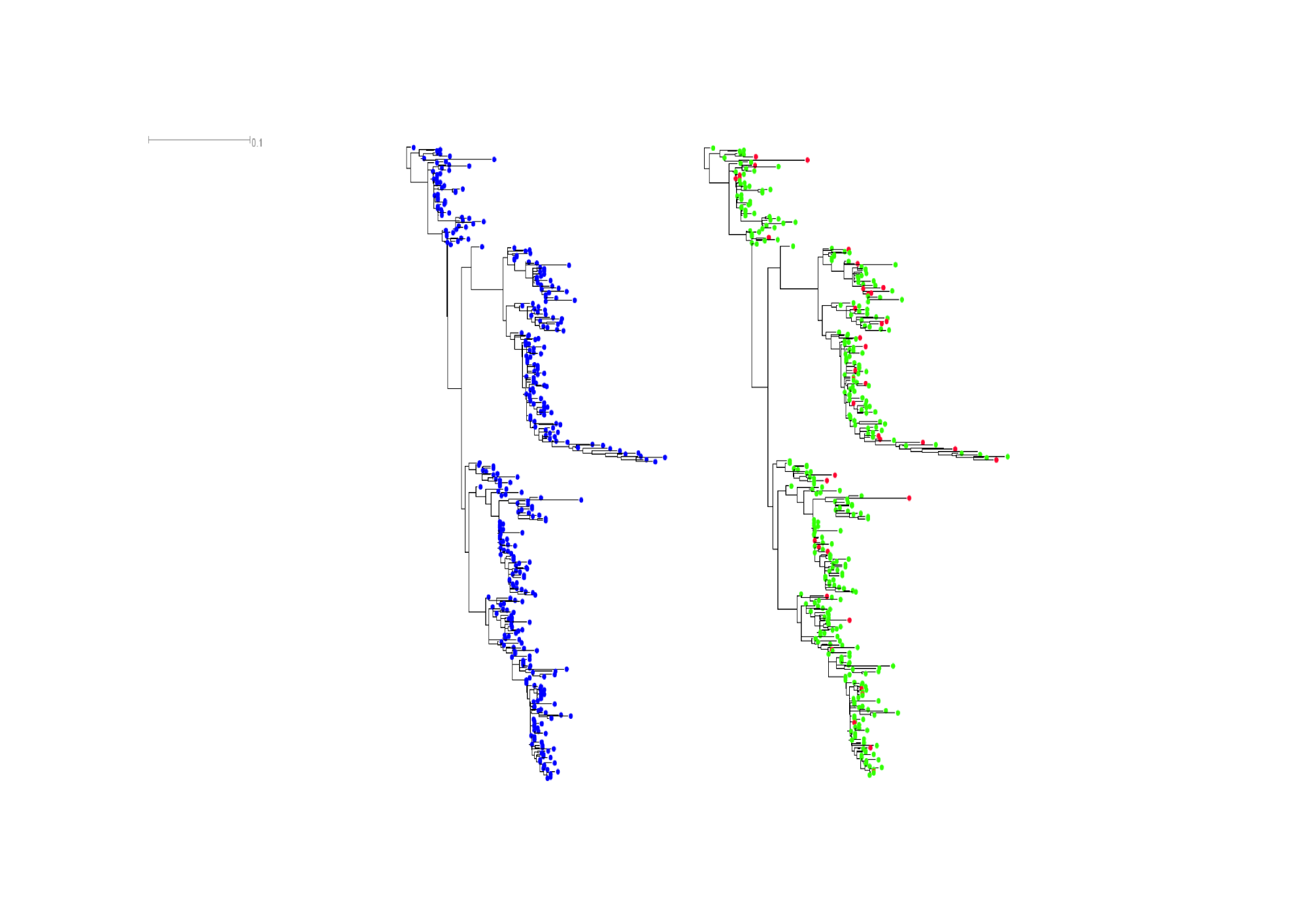

Supplement: Figure S5 — Twin trees obtained from patient 8. The left tree depicts the distribution of viral variants according to the sampling time (blue: first, green: second, pink: third, orange: fourth, gray: fifth); the right tree shows relationships among viral variants according to tropism (X4-using variants: red, R5-using variants: light green). The vertical size of the clusters is proportional to the number of reads in the cluster and the horizontal size of the clusters shows their maximum genetic depth. Branch lengths are proportional to the number of nucleotide substitutions per aligned site (bar = 0.1 substitutions). (TIF) [file pone.0102857.s005.tif]

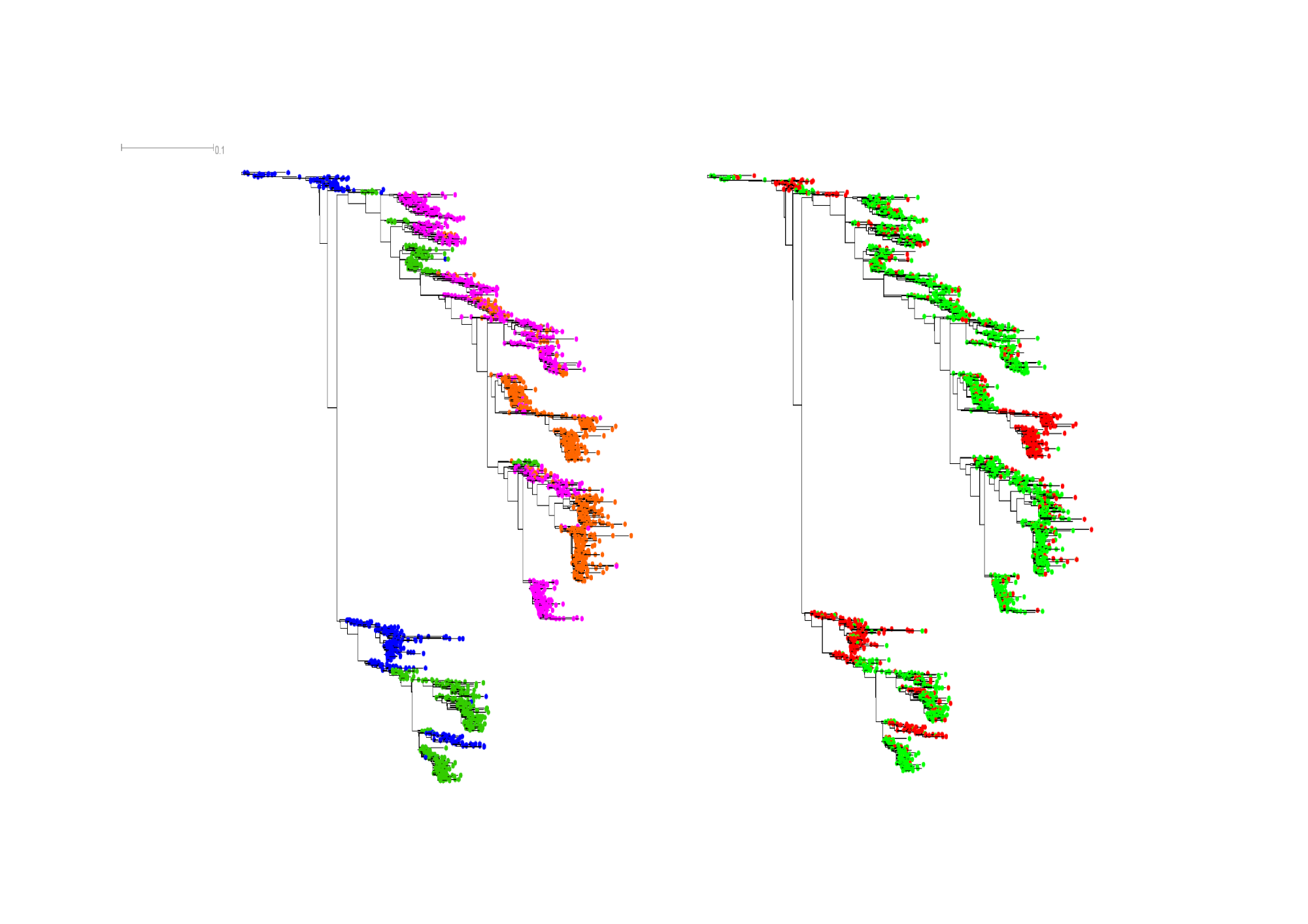

Supplement: Figure S6 — Twin trees obtained from patient 9. The left tree depicts the distribution of viral variants according to the sampling time (blue: first, green: second, pink: third, orange: fourth, gray: fifth); the right tree shows relationships among viral variants according to tropism (X4-using variants: red, R5-using variants: light green). The vertical size of the clusters is proportional to the number of reads in the cluster and the horizontal size of the clusters shows their maximum genetic depth. Branch lengths are proportional to the number of nucleotide substitutions per aligned site (bar = 0.1 substitutions). (TIF) [file pone.0102857.s006.tif]

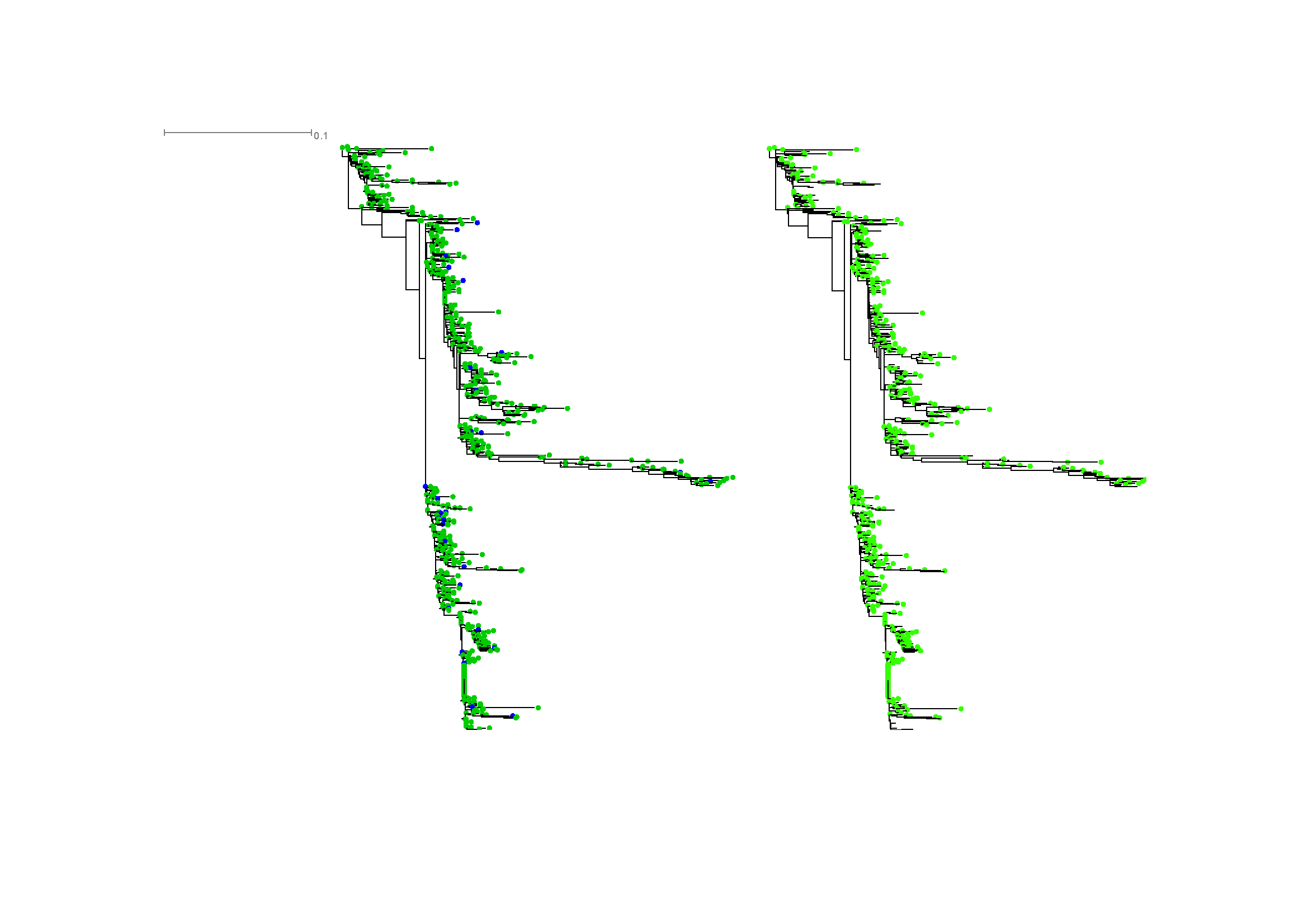

Supplement: Figure S7 — Twin trees obtained from patient 13. The left tree depicts the distribution of viral variants according to the sampling time (blue: first, green: second, pink: third, orange: fourth, gray: fifth); the right tree shows relationships among viral variants according to tropism (X4-using variants: red, R5-using variants: light green). The vertical size of the clusters is proportional to the number of reads in the cluster and the horizontal size of the clusters shows their maximum genetic depth. Branch lengths are proportional to the number of nucleotide substitutions per aligned site (bar = 0.1 substitutions). (TIF) [file pone.0102857.s007.tif]

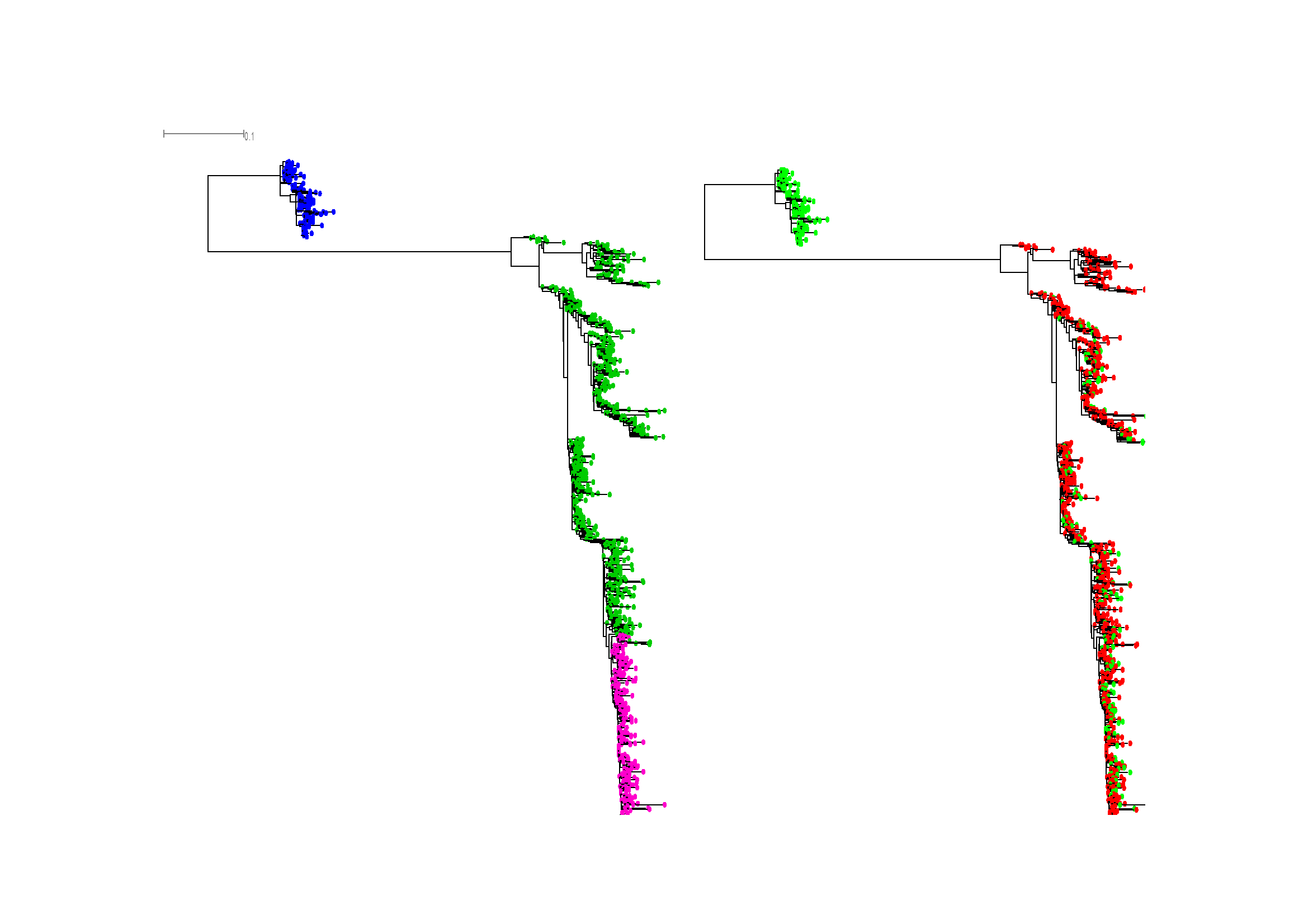

Supplement: Figure S8 — Twin trees obtained from patient 14. The left tree depicts the distribution of viral variants according to the sampling time (blue: first, green: second, pink: third, orange: fourth, gray: fifth); the right tree shows relationships among viral variants according to tropism (X4-using variants: red, R5-using variants: light green). The vertical size of the clusters is proportional to the number of reads in the cluster and the horizontal size of the clusters shows their maximum genetic depth. Branch lengths are proportional to the number of nucleotide substitutions per aligned site (bar = 0.1 substitutions). (TIF) [file pone.0102857.s008.tif]

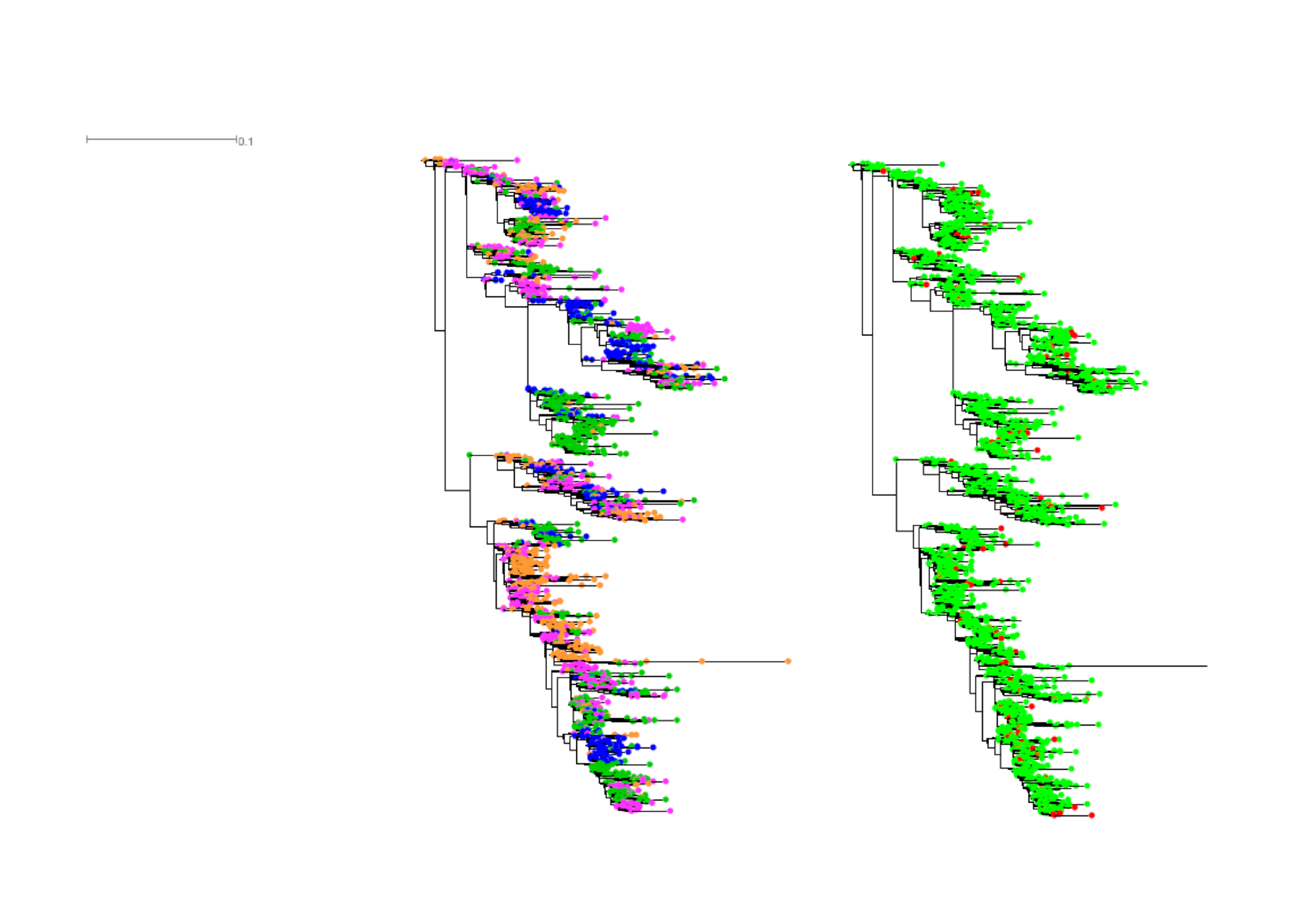

Supplement: Figure S9 — Twin trees obtained from patient 15. The left tree depicts the distribution of viral variants according to the sampling time (blue: first, green: second, pink: third, orange: fourth, gray: fifth); the right tree shows relationships among viral variants according to tropism (X4-using variants: red, R5-using variants: light green). The vertical size of the clusters is proportional to the number of reads in the cluster and the horizontal size of the clusters shows their maximum genetic depth. Branch lengths are proportional to the number of nucleotide substitutions per aligned site (bar = 0.1 substitutions). (TIF) [file pone.0102857.s009.tif]

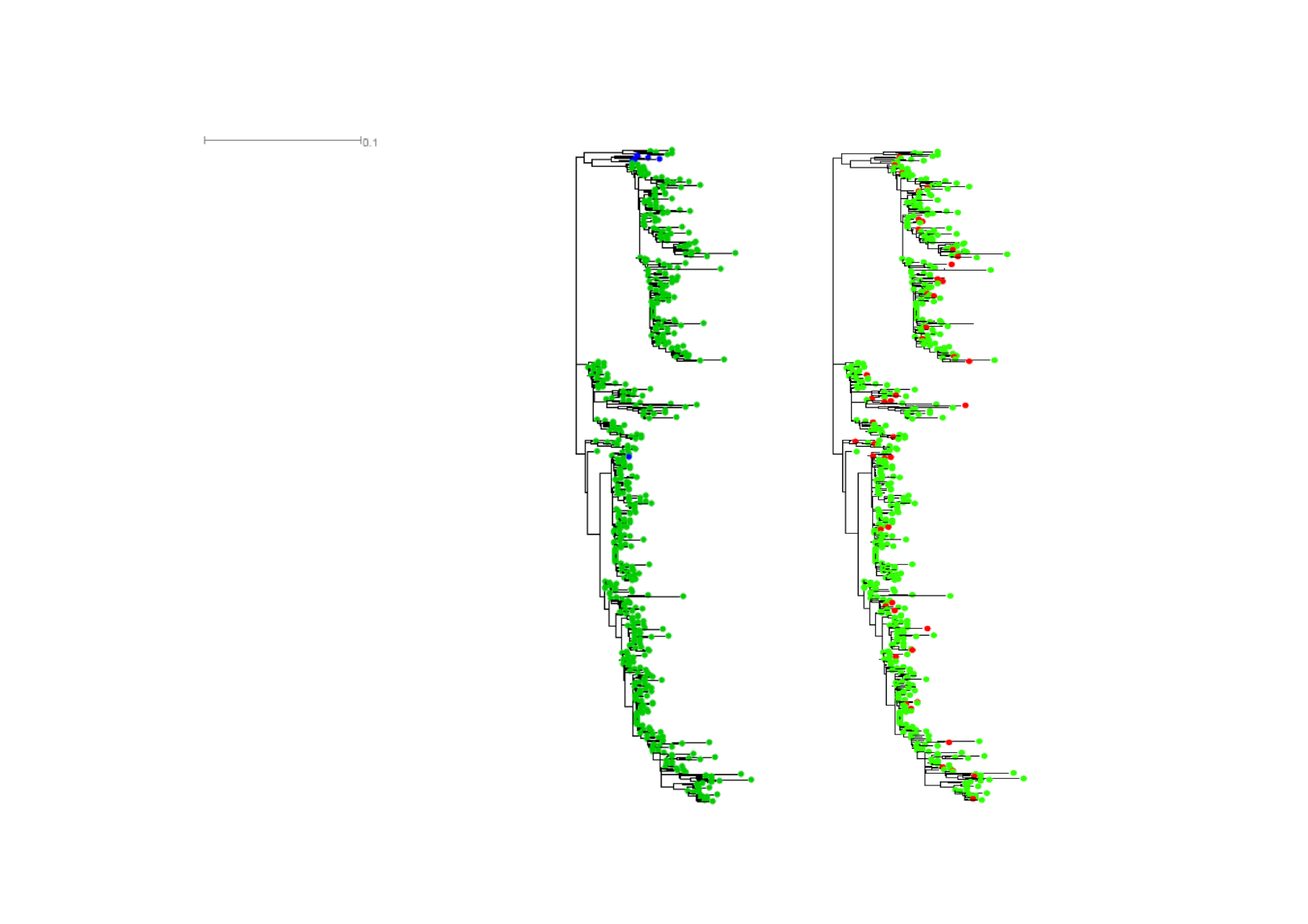

Supplement: Figure S10 — Twin trees obtained from patient 17. The left tree depicts the distribution of viral variants according to the sampling time (blue: first, green: second, pink: third, orange: fourth, gray: fifth); the right tree shows relationships among viral variants according to tropism (X4-using variants: red, R5-using variants: light green). The vertical size of the clusters is proportional to the number of reads in the cluster and the horizontal size of the clusters shows their maximum genetic depth. Branch lengths are proportional to the number of nucleotide substitutions per aligned site (bar = 0.1 substitutions). (TIF) [file pone.0102857.s010.tif]

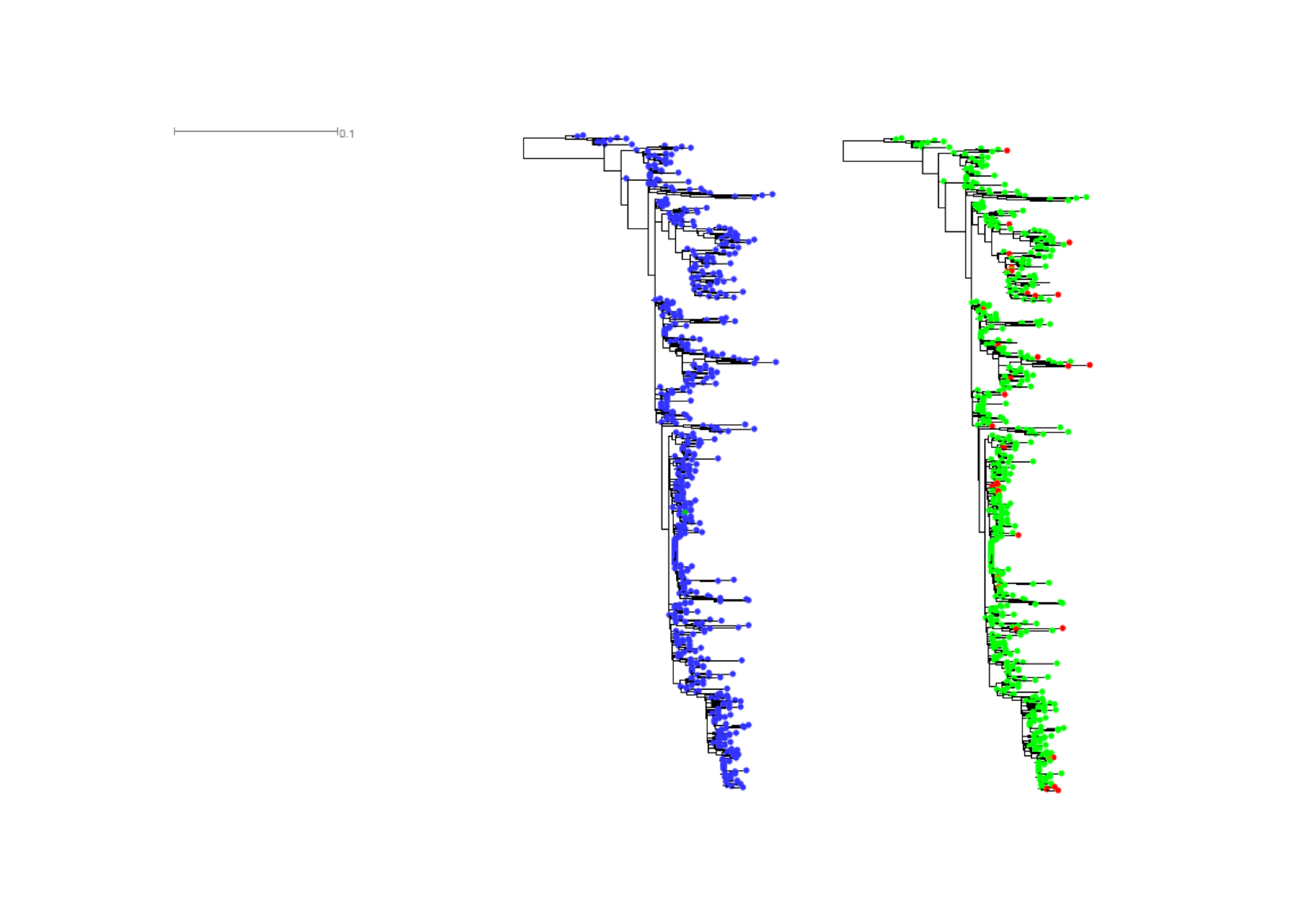

Supplement: Figure S11 — Twin trees obtained from patient 18. The left tree depicts the distribution of viral variants according to the sampling time (blue: first, green: second, pink: third, orange: fourth, gray: fifth); the right tree shows relationships among viral variants according to tropism (X4-using variants: red, R5-using variants: light green). The vertical size of the clusters is proportional to the number of reads in the cluster and the horizontal size of the clusters shows their maximum genetic depth. Branch lengths are proportional to the number of nucleotide substitutions per aligned site (bar = 0.1 substitutions). (TIF) [file pone.0102857.s011.tif]

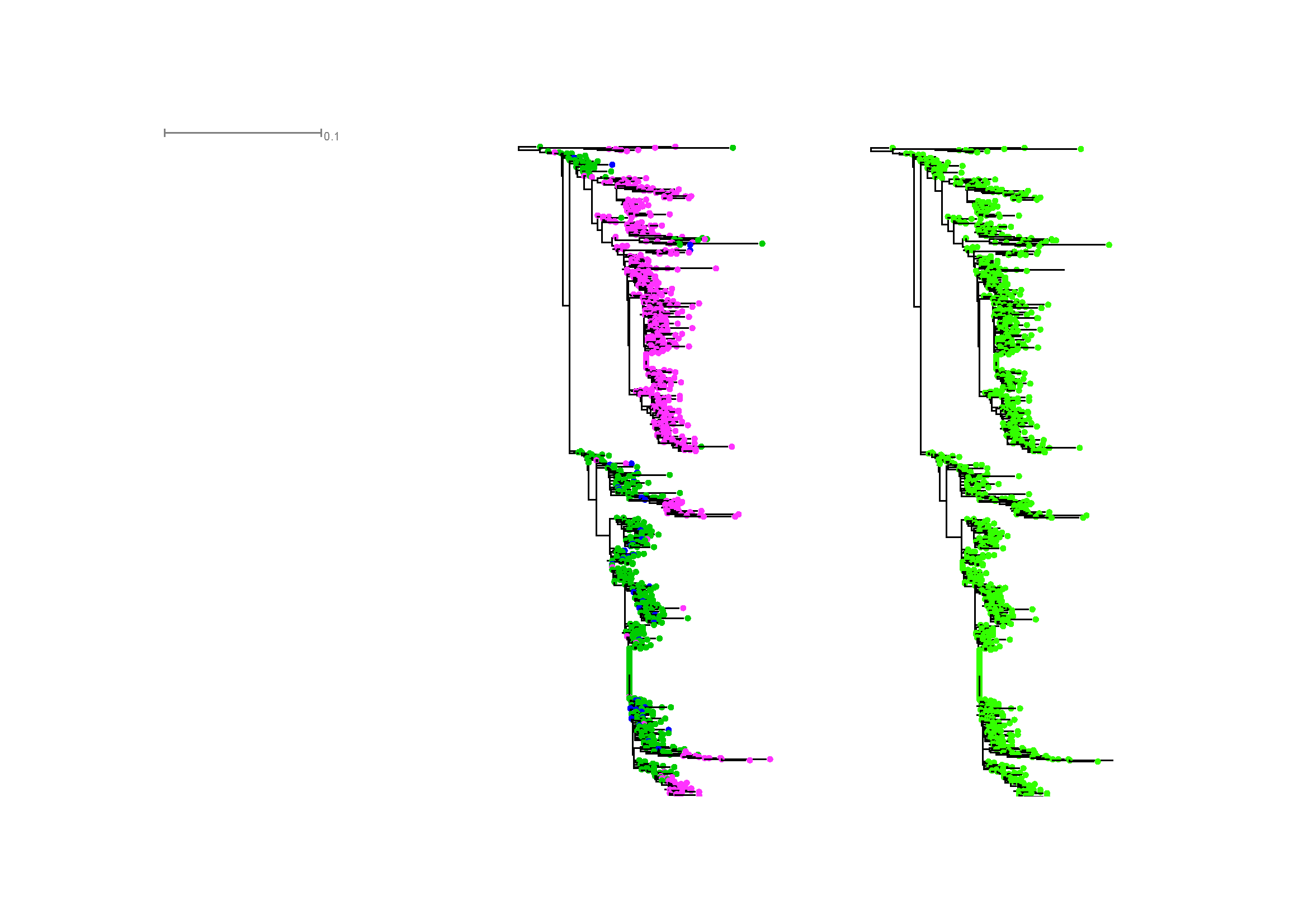

Supplement: Figure S12 — Twin trees obtained from patient 21. The left tree depicts the distribution of viral variants according to the sampling time (blue: first, green: second, pink: third, orange: fourth, gray: fifth); the right tree shows relationships among viral variants according to tropism (X4-using variants: red, R5-using variants: light green). The vertical size of the clusters is proportional to the number of reads in the cluster and the horizontal size of the clusters shows their maximum genetic depth. Branch lengths are proportional to the number of nucleotide substitutions per aligned site (bar = 0.1 substitutions). (TIF) [file pone.0102857.s012.tif]

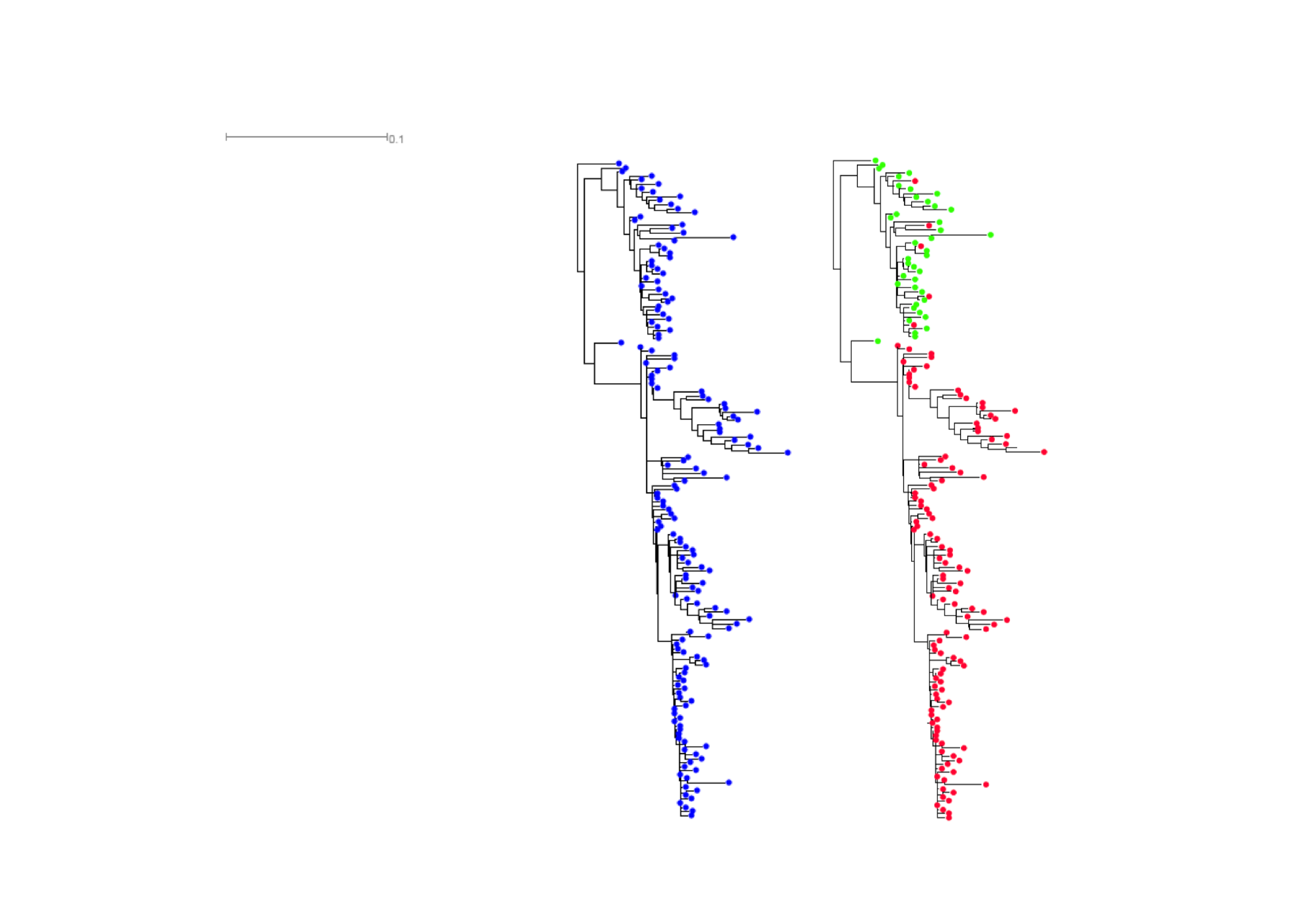

Supplement: Figure S13 — Twin trees obtained from patient 23. The left tree depicts the distribution of viral variants according to the sampling time (blue: first, green: second, pink: third, orange: fourth, gray: fifth); the right tree shows relationships among viral variants according to tropism (X4-using variants: red, R5-using variants: light green). The vertical size of the clusters is proportional to the number of reads in the cluster and the horizontal size of the clusters shows their maximum genetic depth. Branch lengths are proportional to the number of nucleotide substitutions per aligned site (bar = 0.1 substitutions). (TIF) [file pone.0102857.s013.tif]

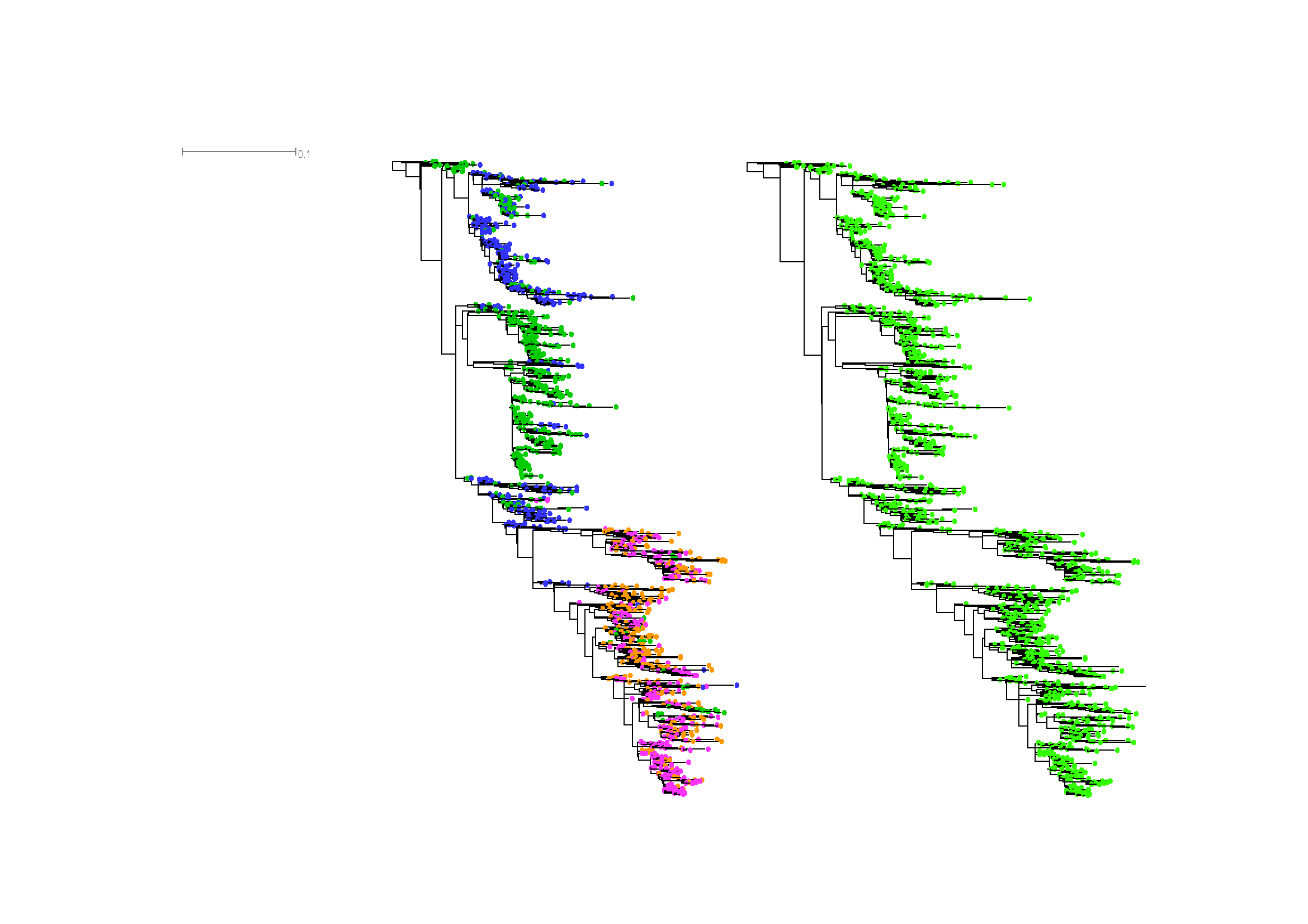

Supplement: Figure S14 — Twin trees obtained from patient 24. The left tree depicts the distribution of viral variants according to the sampling time (blue: first, green: second, pink: third, orange: fourth, gray: fifth); the right tree shows relationships among viral variants according to tropism (X4-using variants: red, R5-using variants: light green). The vertical size of the clusters is proportional to the number of reads in the cluster and the horizontal size of the clusters shows their maximum genetic depth. Branch lengths are proportional to the number of nucleotide substitutions per aligned site (bar = 0.1 substitutions). (TIF) [file pone.0102857.s014.tif]

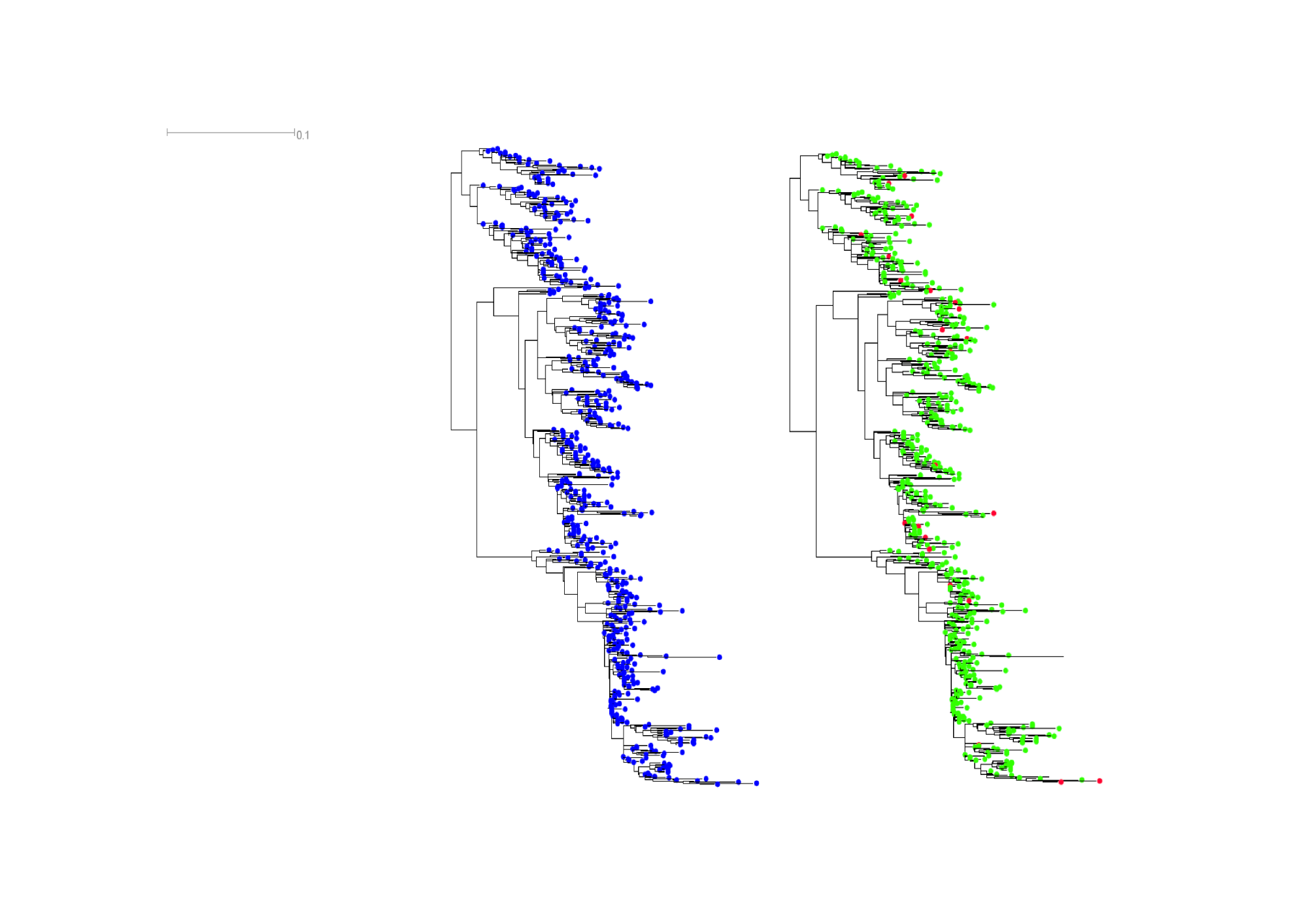

Supplement: Figure S15 — Twin trees obtained from patient 26. The left tree depicts the distribution of viral variants according to the sampling time (blue: first, green: second, pink: third, orange: fourth, gray: fifth); the right tree shows relationships among viral variants according to tropism (X4-using variants: red, R5-using variants: light green). The vertical size of the clusters is proportional to the number of reads in the cluster and the horizontal size of the clusters shows their maximum genetic depth. Branch lengths are proportional to the number of nucleotide substitutions per aligned site (bar = 0.1 substitutions). (TIF) [file pone.0102857.s015.tif]

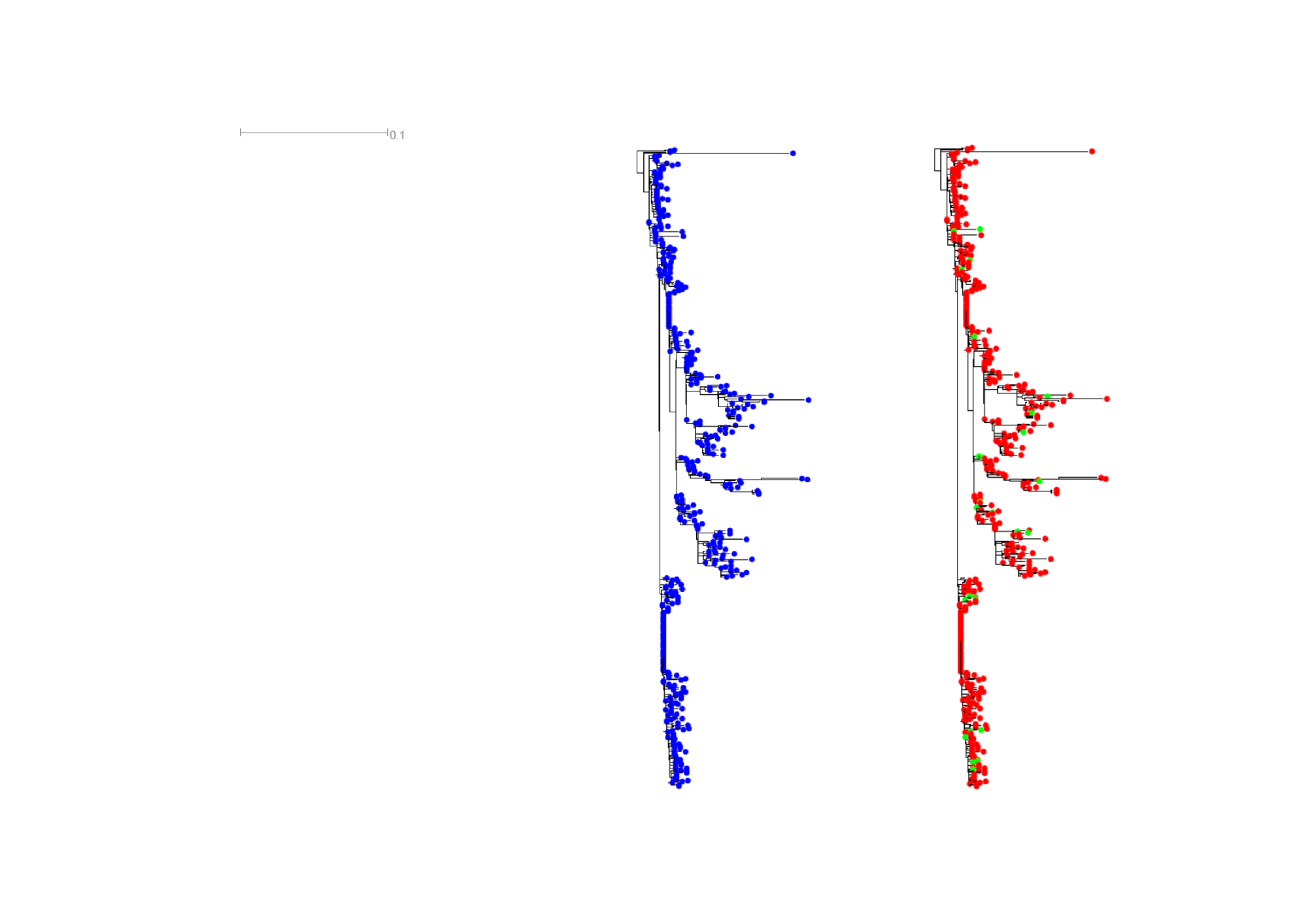

Supplement: Figure S16 — Twin trees obtained from patient 27. The left tree depicts the distribution of viral variants according to the sampling time (blue: first, green: second, pink: third, orange: fourth, gray: fifth); the right tree shows relationships among viral variants according to tropism (X4-using variants: red, R5-using variants: light green). The vertical size of the clusters is proportional to the number of reads in the cluster and the horizontal size of the clusters shows their maximum genetic depth. Branch lengths are proportional to the number of nucleotide substitutions per aligned site (bar = 0.1 substitutions). (TIF) [file pone.0102857.s016.tif]

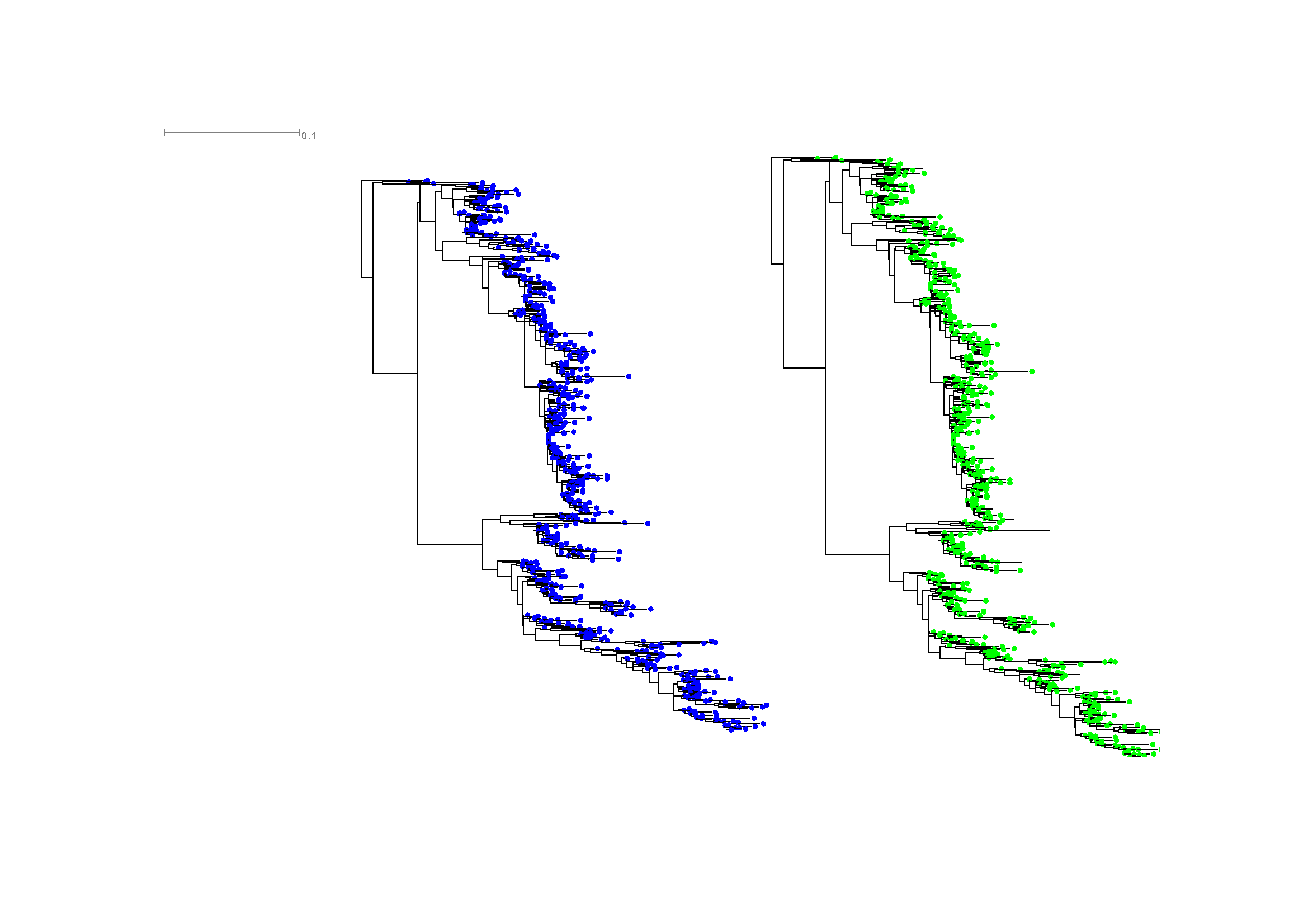

Supplement: Figure S17 — Twin trees obtained from patient 28. The left tree depicts the distribution of viral variants according to the sampling time (blue: first, green: second, pink: third, orange: fourth, gray: fifth); the right tree shows relationships among viral variants according to tropism (X4-using variants: red, R5-using variants: light green). The vertical size of the clusters is proportional to the number of reads in the cluster and the horizontal size of the clusters shows their maximum genetic depth. Branch lengths are proportional to the number of nucleotide substitutions per aligned site (bar = 0.1 substitutions). (TIF) [file pone.0102857.s017.tif]

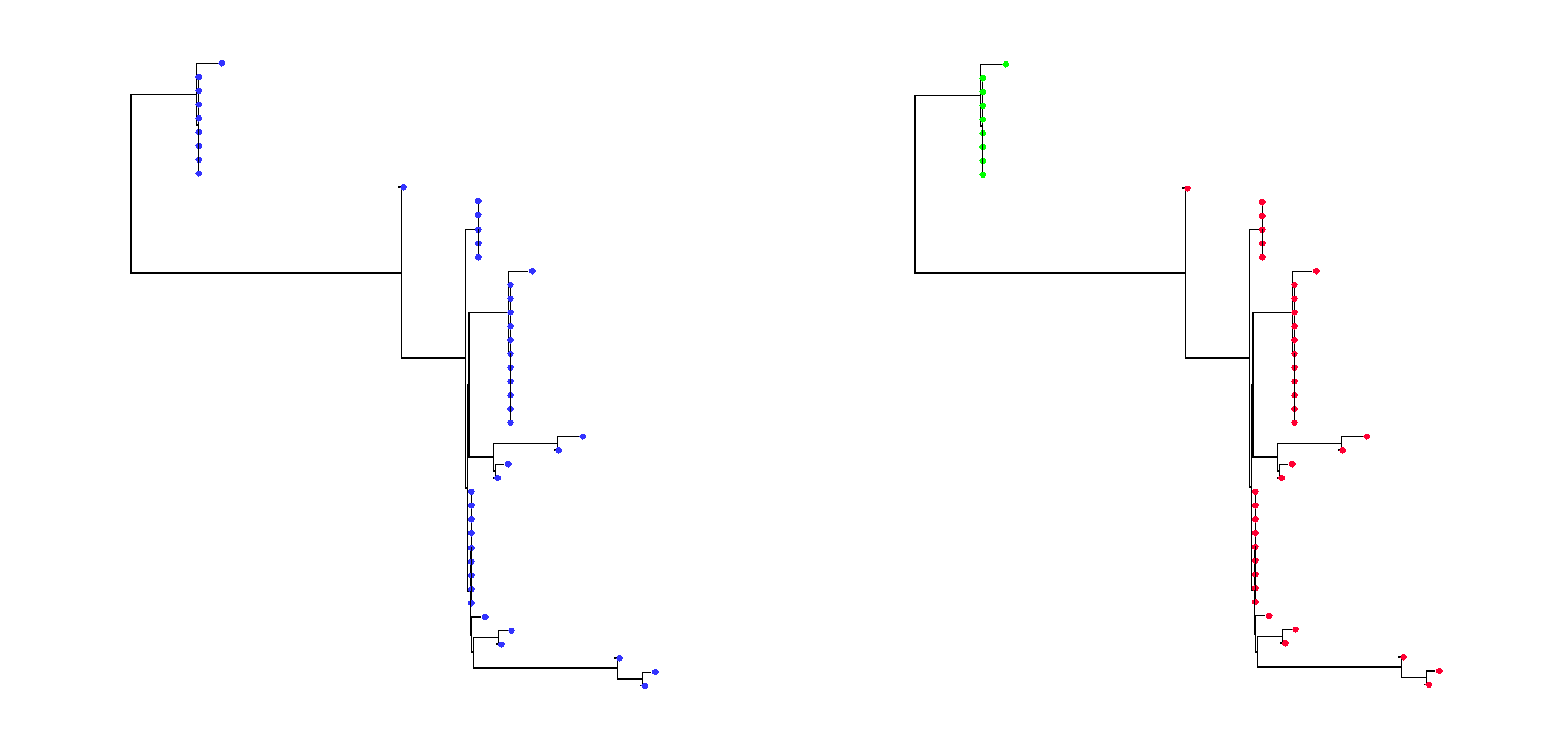

Supplement: Figure S18 — Phylogenetic analysis of Patient 1 Kec haplotypes. The trees in left and right panels are twin trees on which isolation time and tropism, respectively, were mapped. Color codes are as in Figure 2. (TIF) [file pone.0102857.s018.tif]

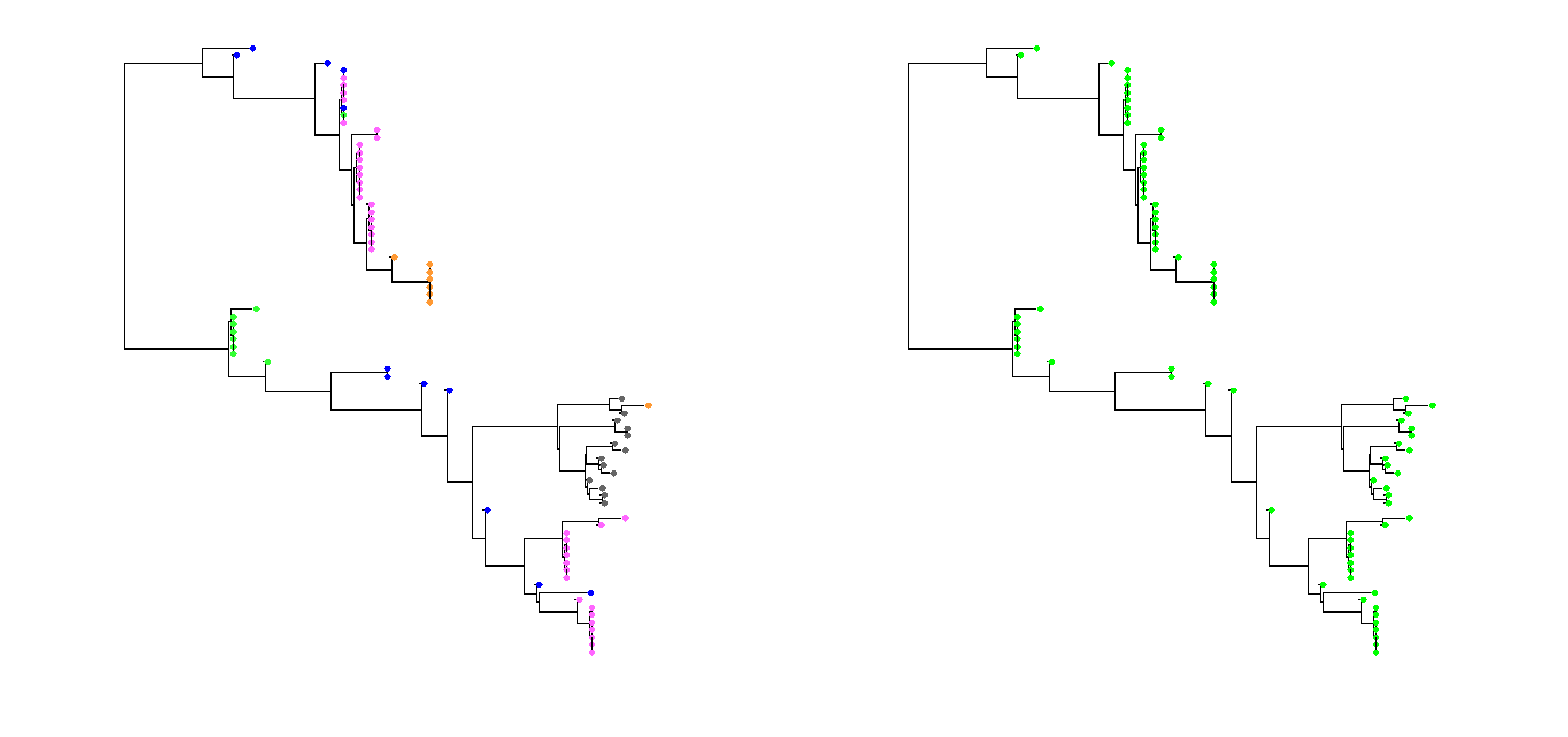

Supplement: Figure S19 — Phylogenetic analysis of Patient 3 Kec haplotypes. The trees in left and right panels are twin trees on which isolation time and tropism, respectively, were mapped. Color codes are as in Figure 2. (TIF) [file pone.0102857.s019.tif]

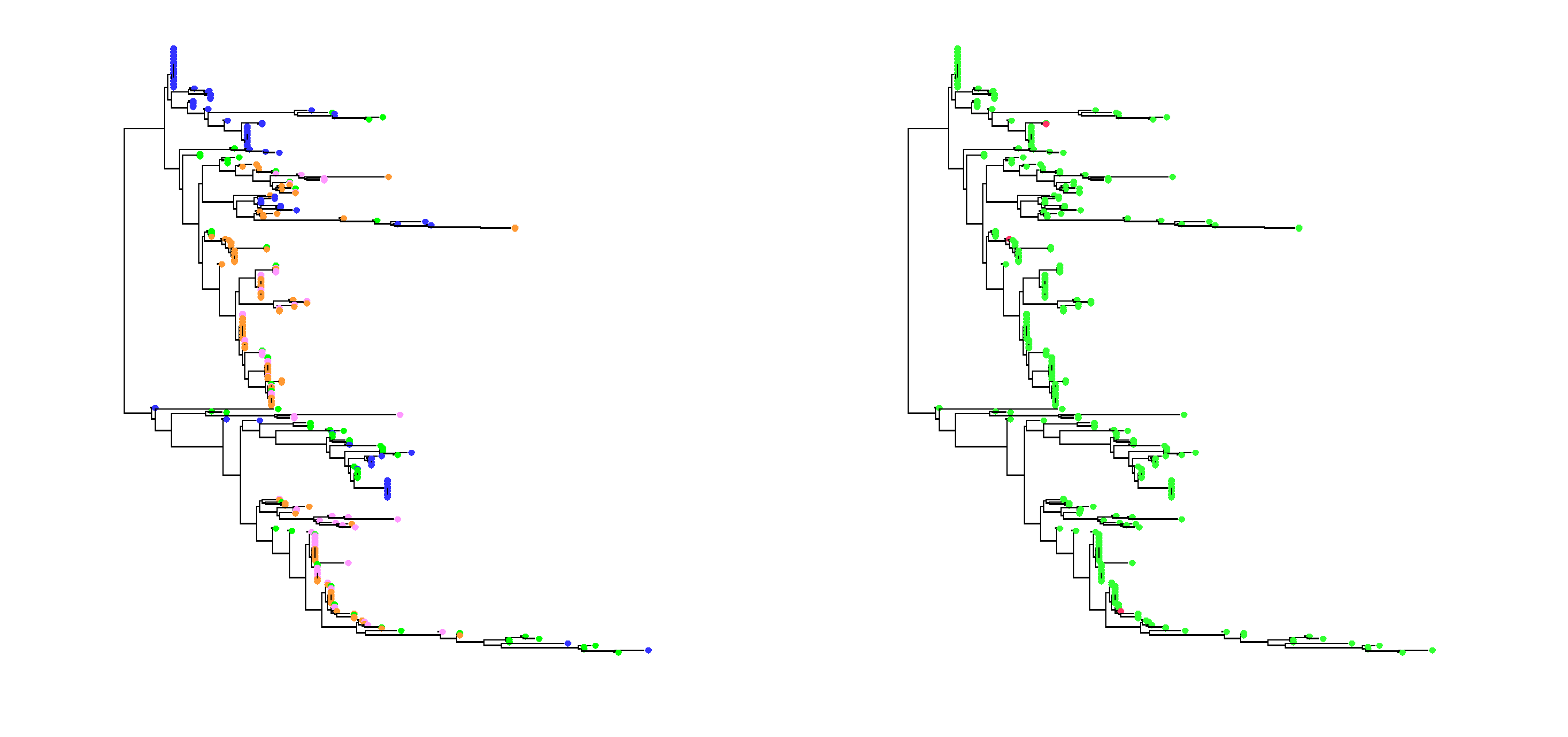

Supplement: Figure S20 — Phylogenetic analysis of Patient 6 Kec haplotypes. The trees in left and right panels are twin trees on which isolation time and tropism, respectively, were mapped. Color codes are as in Figure 2. (TIF) [file pone.0102857.s020.tif]

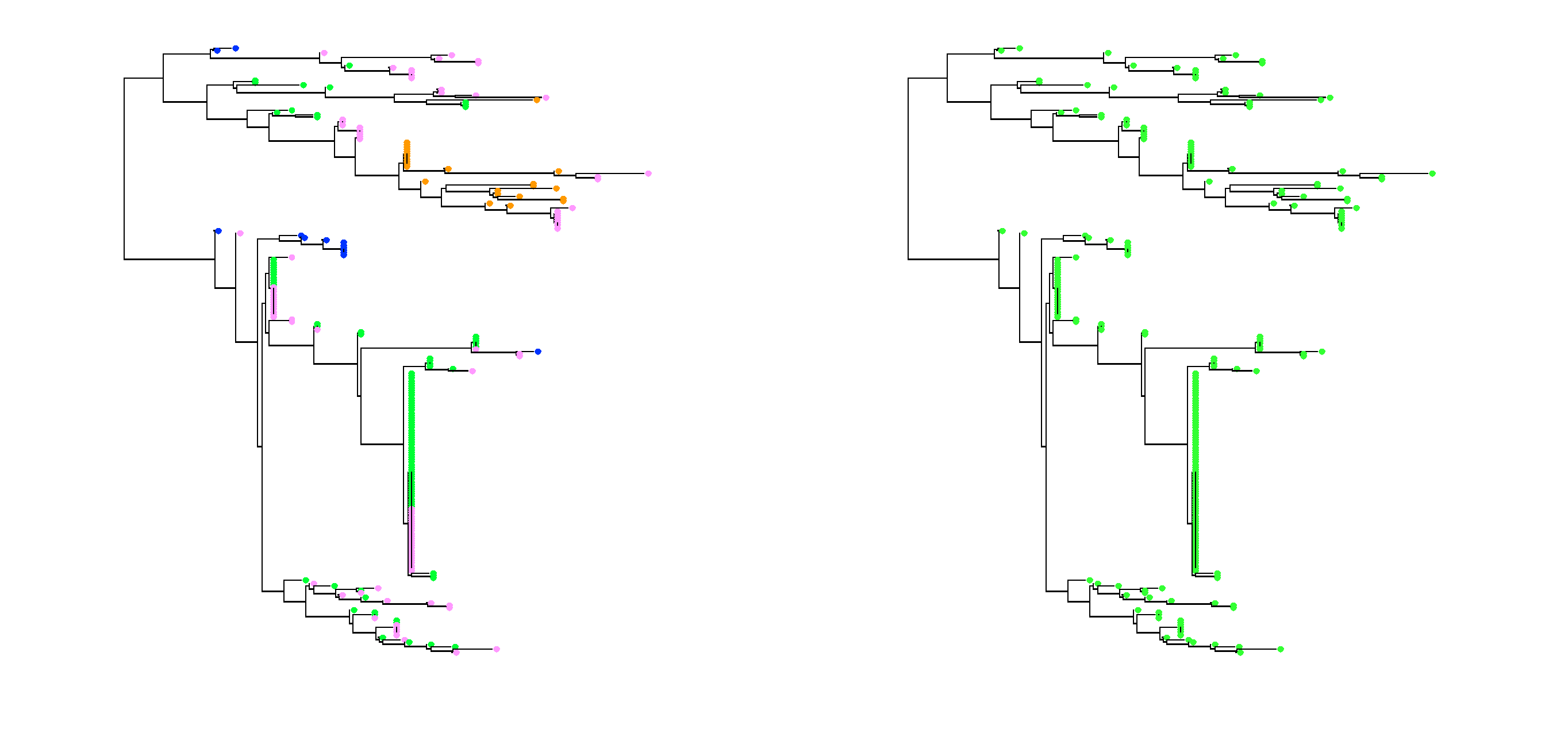

Supplement: Figure S21 — Phylogenetic analysis of Patient 7 Kec haplotypes. The trees in left and right panels are twin trees on which isolation time and tropism, respectively, were mapped. Color codes are as in Figure 2. (TIF) [file pone.0102857.s021.tif]

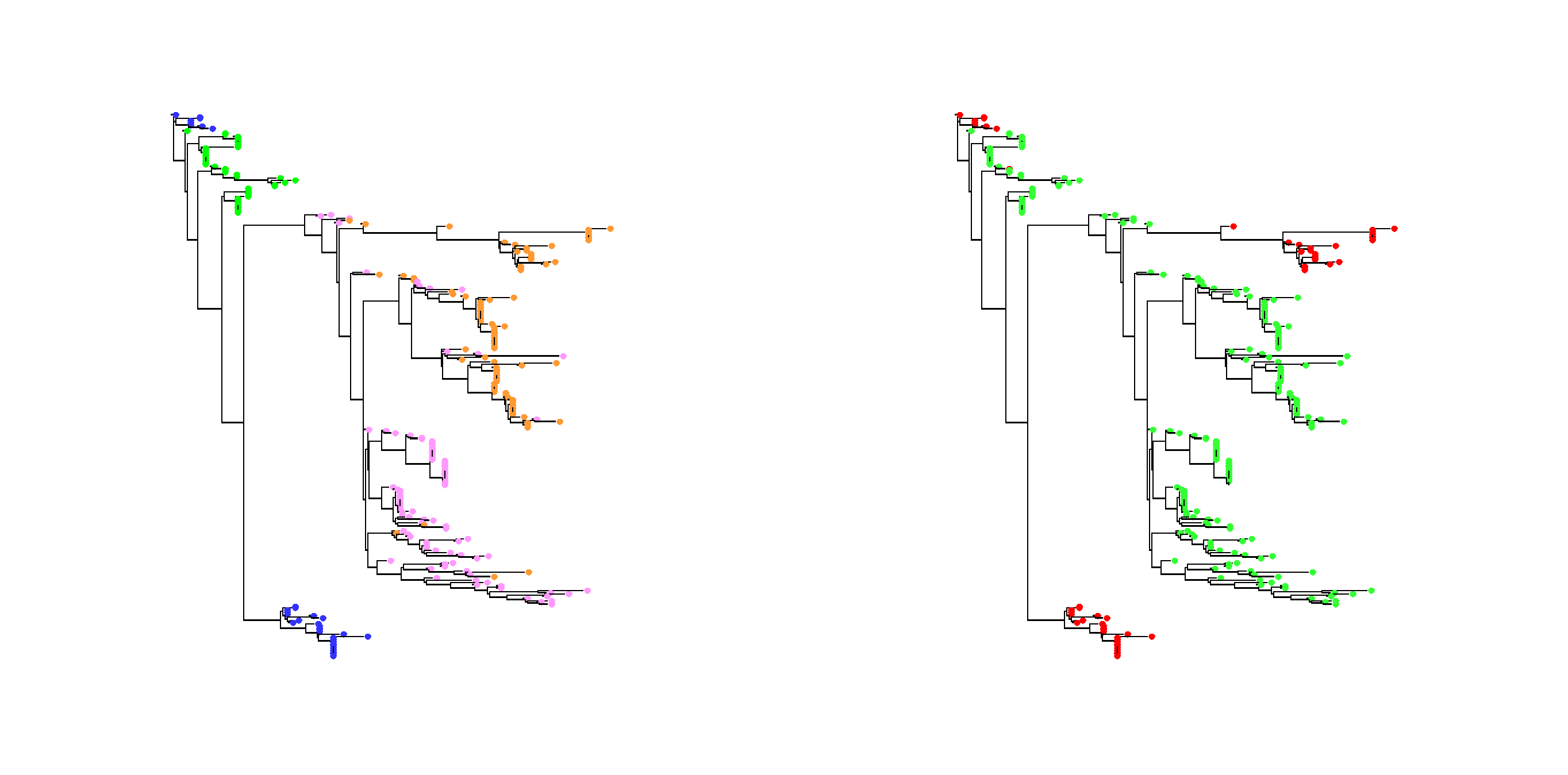

Supplement: Figure S22 — Phylogenetic analysis of Patient 9 Kec haplotypes. The trees in left and right panels are twin trees on which isolation time and tropism, respectively, were mapped. Color codes are as in Figure 2. (TIF) [file pone.0102857.s022.tif]

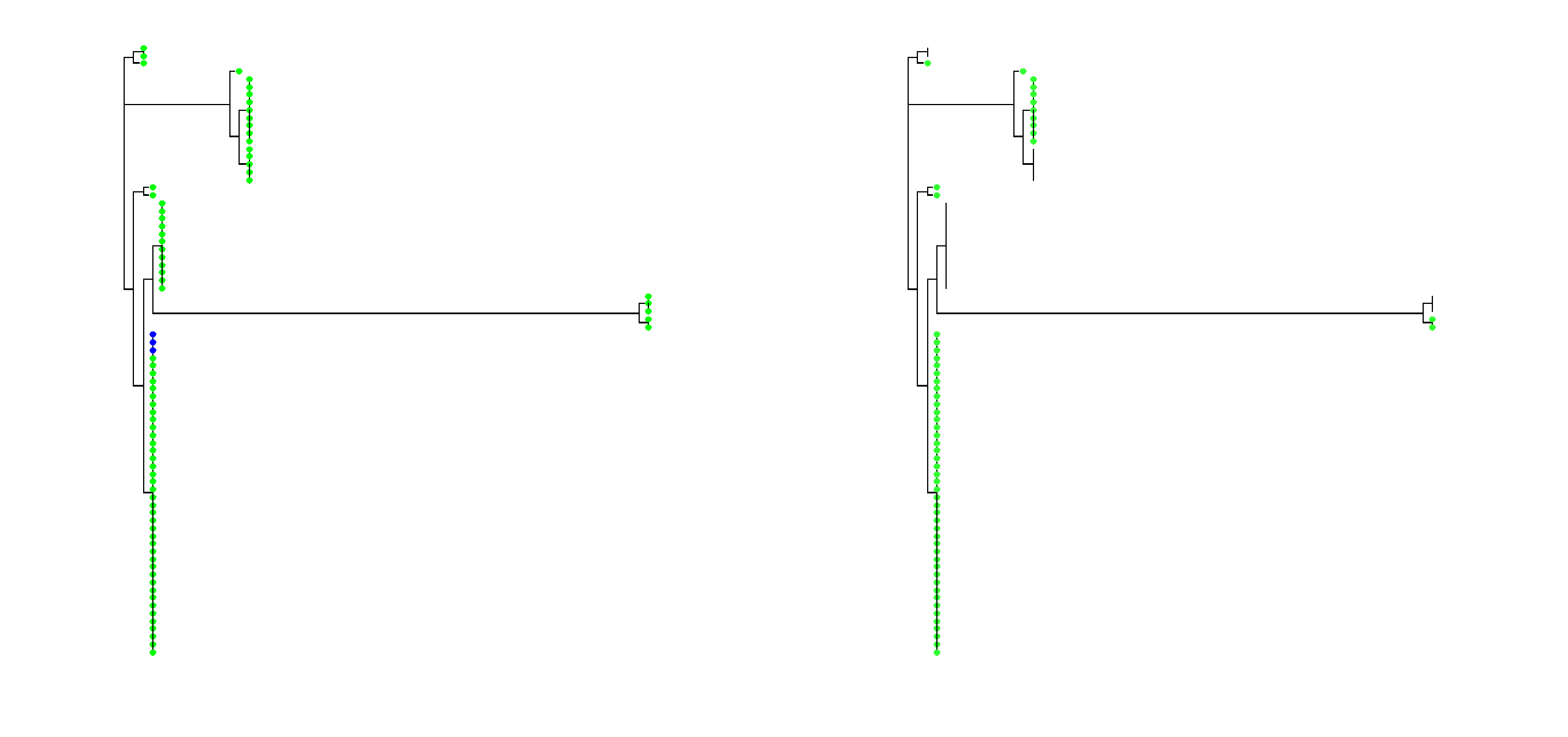

Supplement: Figure S23 — Phylogenetic analysis of Patient 13 Kec haplotypes. The trees in left and right panels are twin trees on which isolation time and tropism, respectively, were mapped. Color codes are as in Figure 2. (TIF) [file pone.0102857.s023.tif]

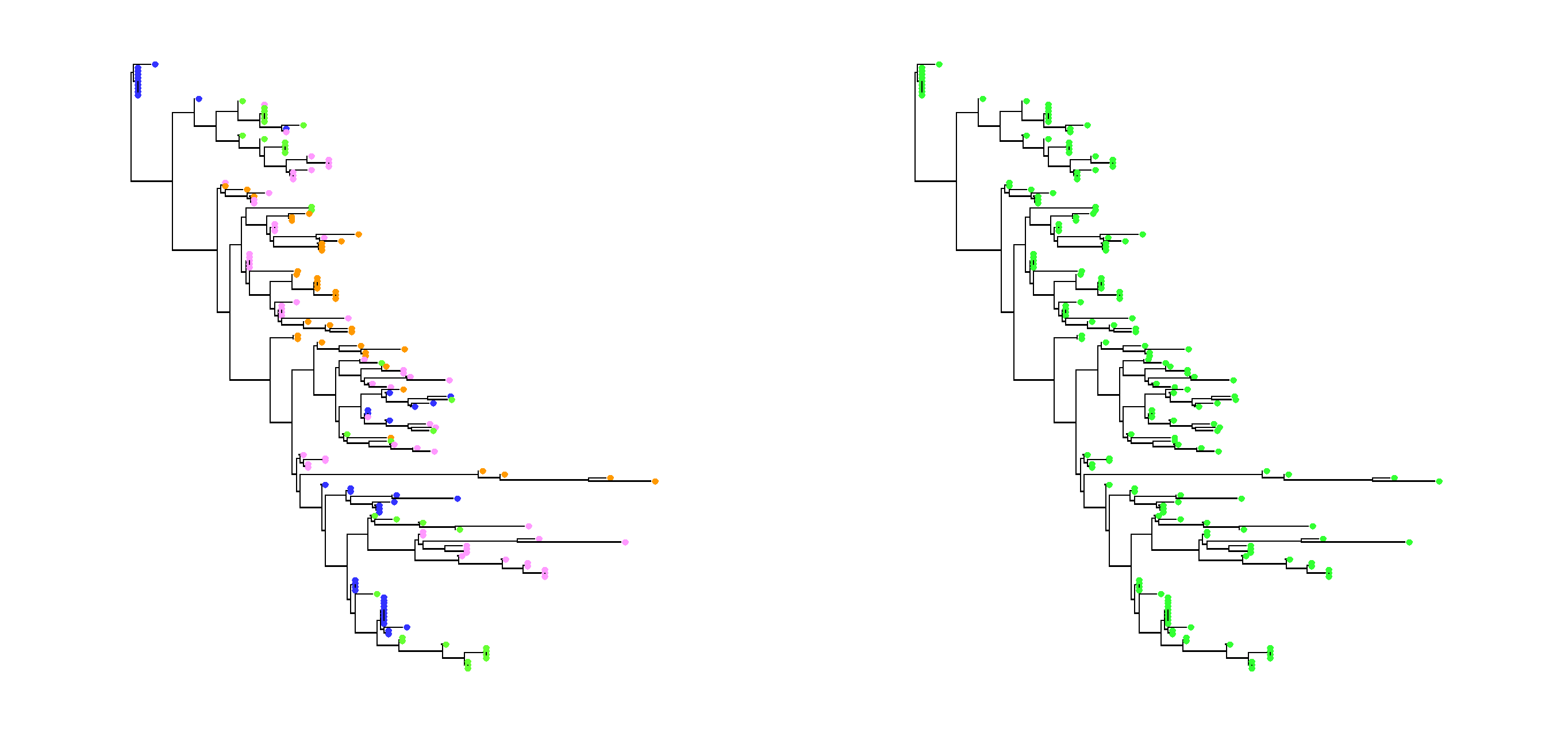

Supplement: Figure S24 — Phylogenetic analysis of Patient 15 Kec haplotypes. The trees in left and right panels are twin trees on which isolation time and tropism, respectively, were mapped. Color codes are as in Figure 2. (TIF) [file pone.0102857.s024.tif]

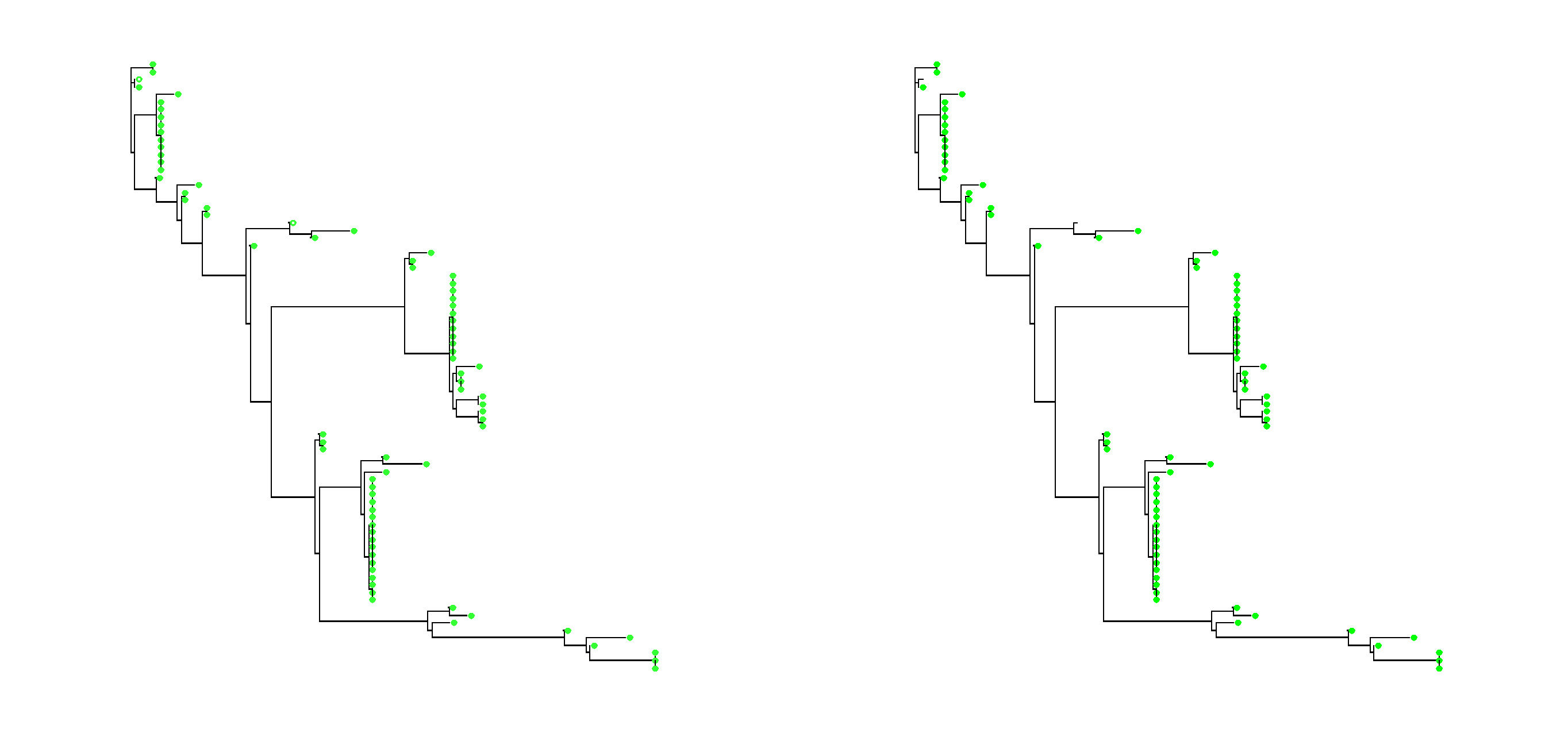

Supplement: Figure S25 — Phylogenetic analysis of Patient 17 Kec haplotypes. The trees in left and right panels are twin trees on which isolation time and tropism, respectively, were mapped. Color codes are as in Figure 2. (TIF) [file pone.0102857.s025.tif]

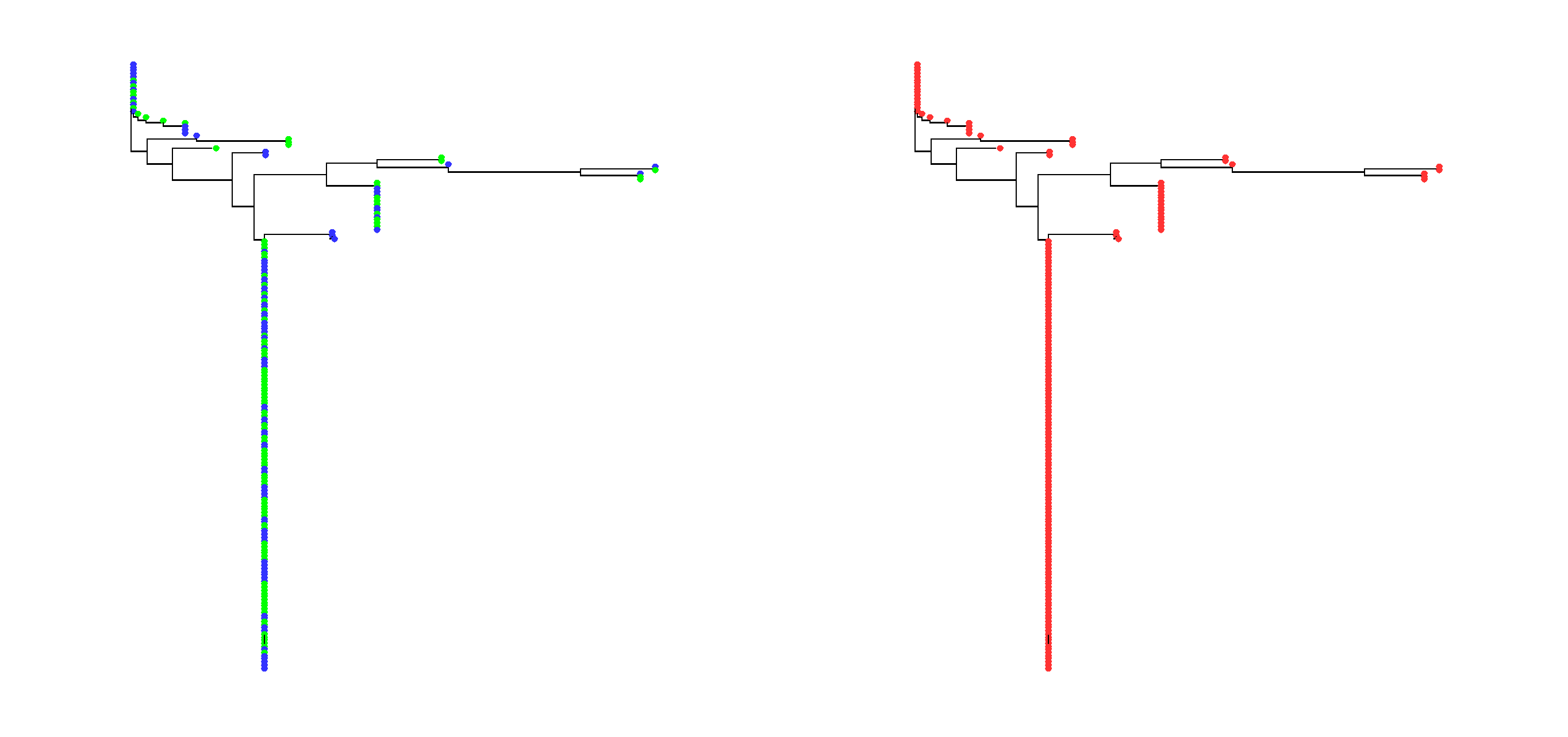

Supplement: Figure S26 — Phylogenetic analysis of Patient 20 Kec haplotypes. The trees in left and right panels are twin trees on which isolation time and tropism, respectively, were mapped. Color codes are as in Figure 2. (TIF) [file pone.0102857.s026.tif]

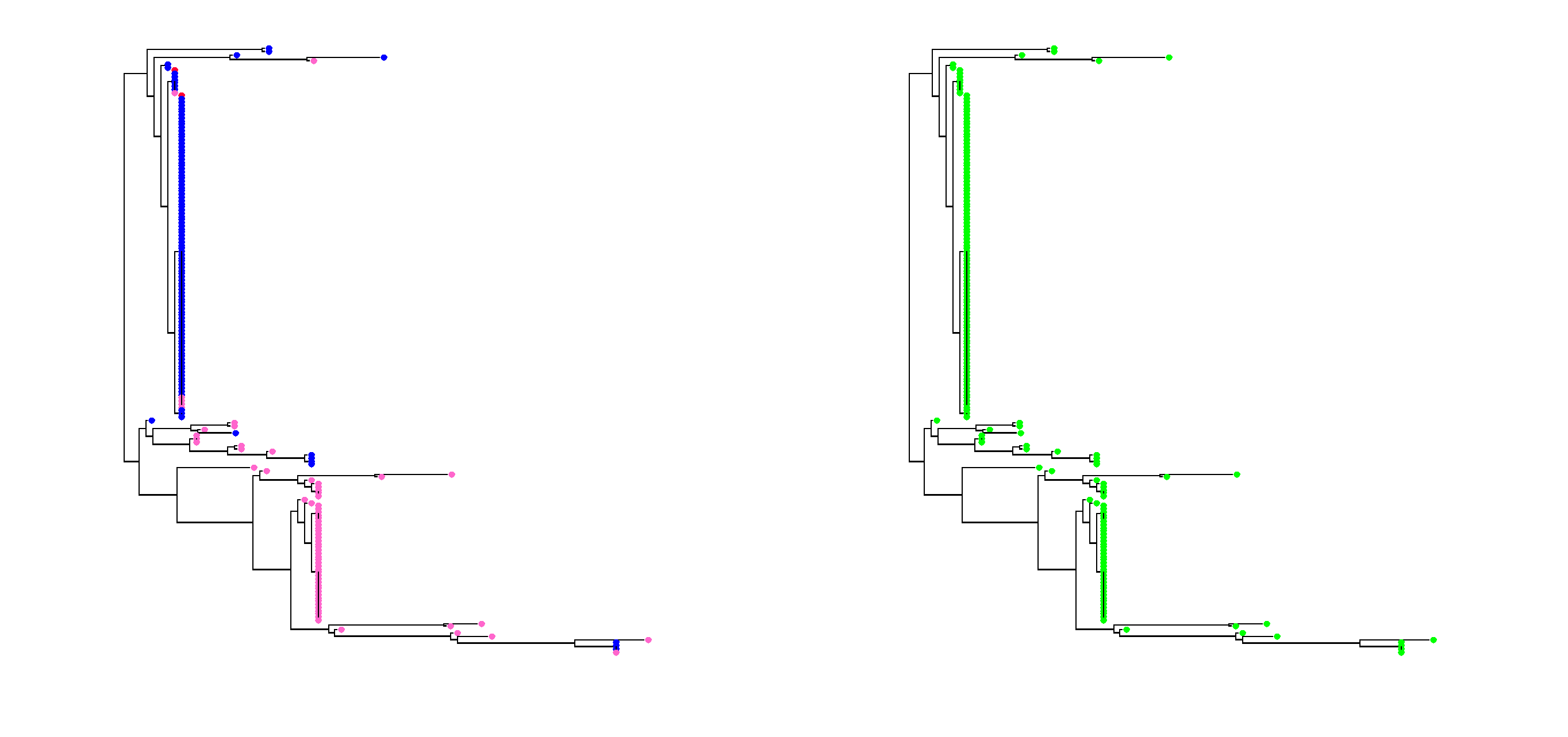

Supplement: Figure S27 — Phylogenetic analysis of Patient 21 Kec haplotypes. The trees in left and right panels are twin trees on which isolation time and tropism, respectively, were mapped. Color codes are as in Figure 2. (TIF) [file pone.0102857.s027.tif]

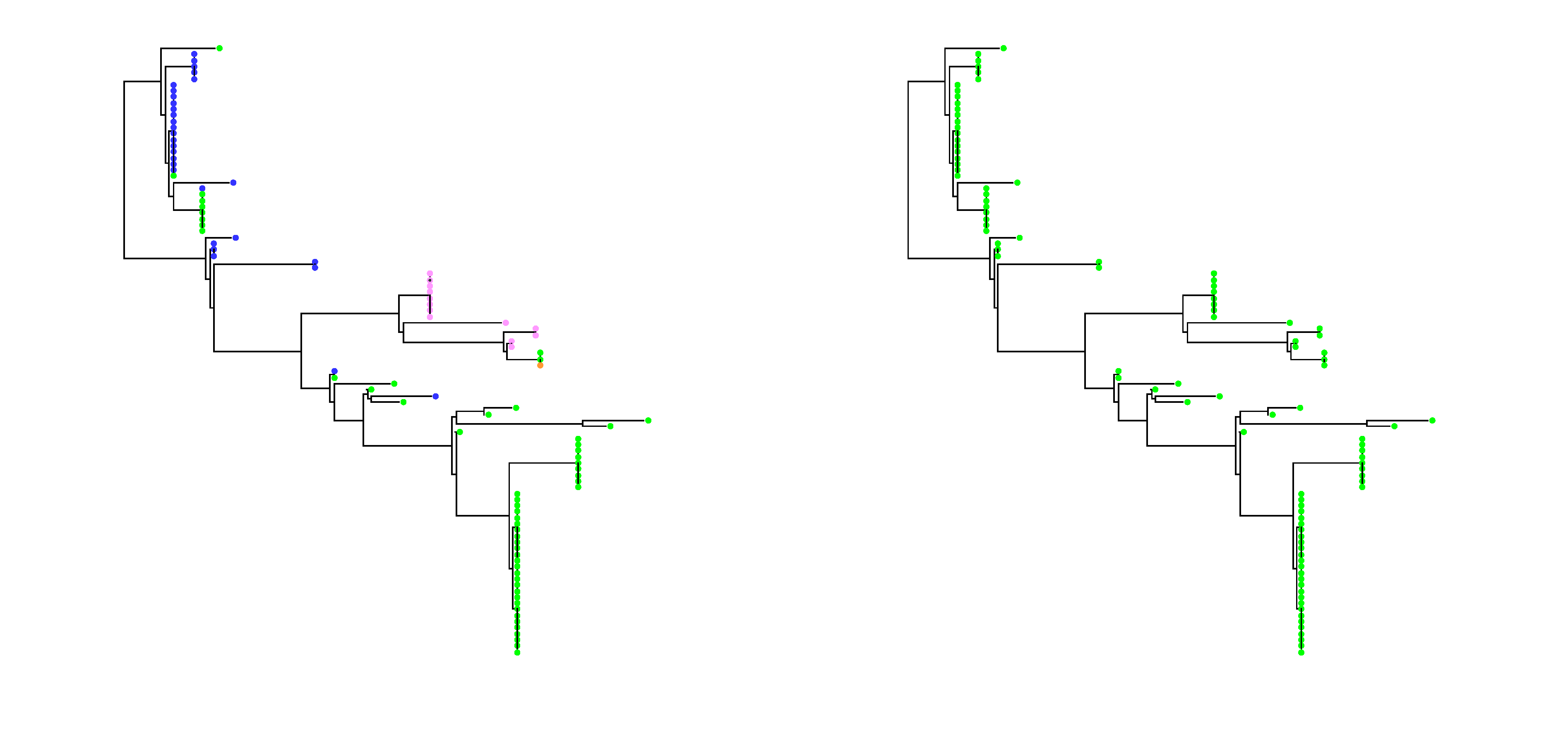

Supplement: Figure S28 — Phylogenetic analysis of Patient 24 Kec haplotypes. The trees in left and right panels are twin trees on which isolation time and tropism, respectively, were mapped. Color codes are as in Figure 2. (TIF) [file pone.0102857.s028.tif]

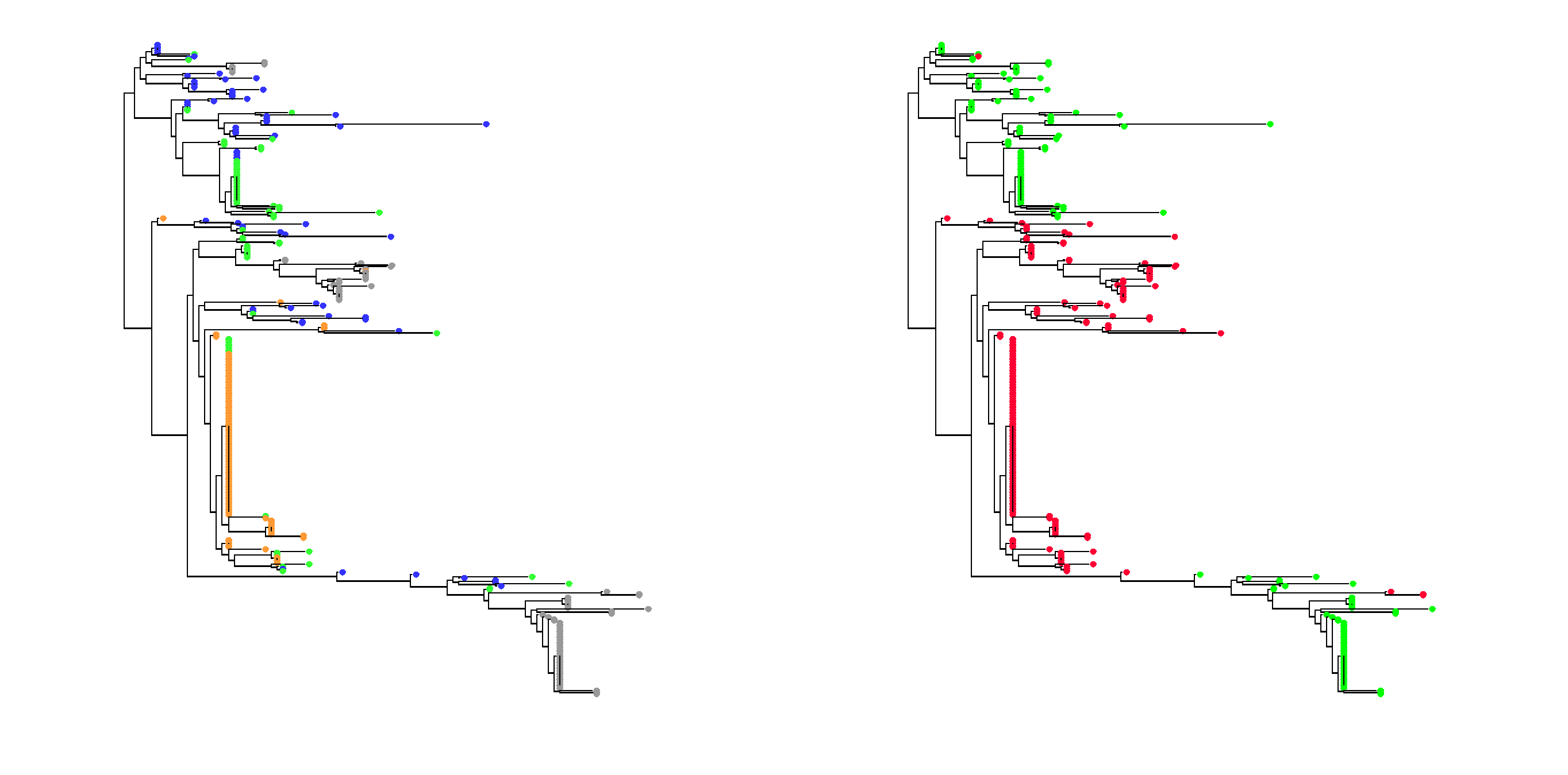

Supplement: Figure S29 — Phylogenetic analysis of Patient 25 Kec haplotypes. The trees in left and right panels are twin trees on which isolation time and tropism, respectively, were mapped. Color codes are as in Figure 2. (TIF) [file pone.0102857.s029.tif]

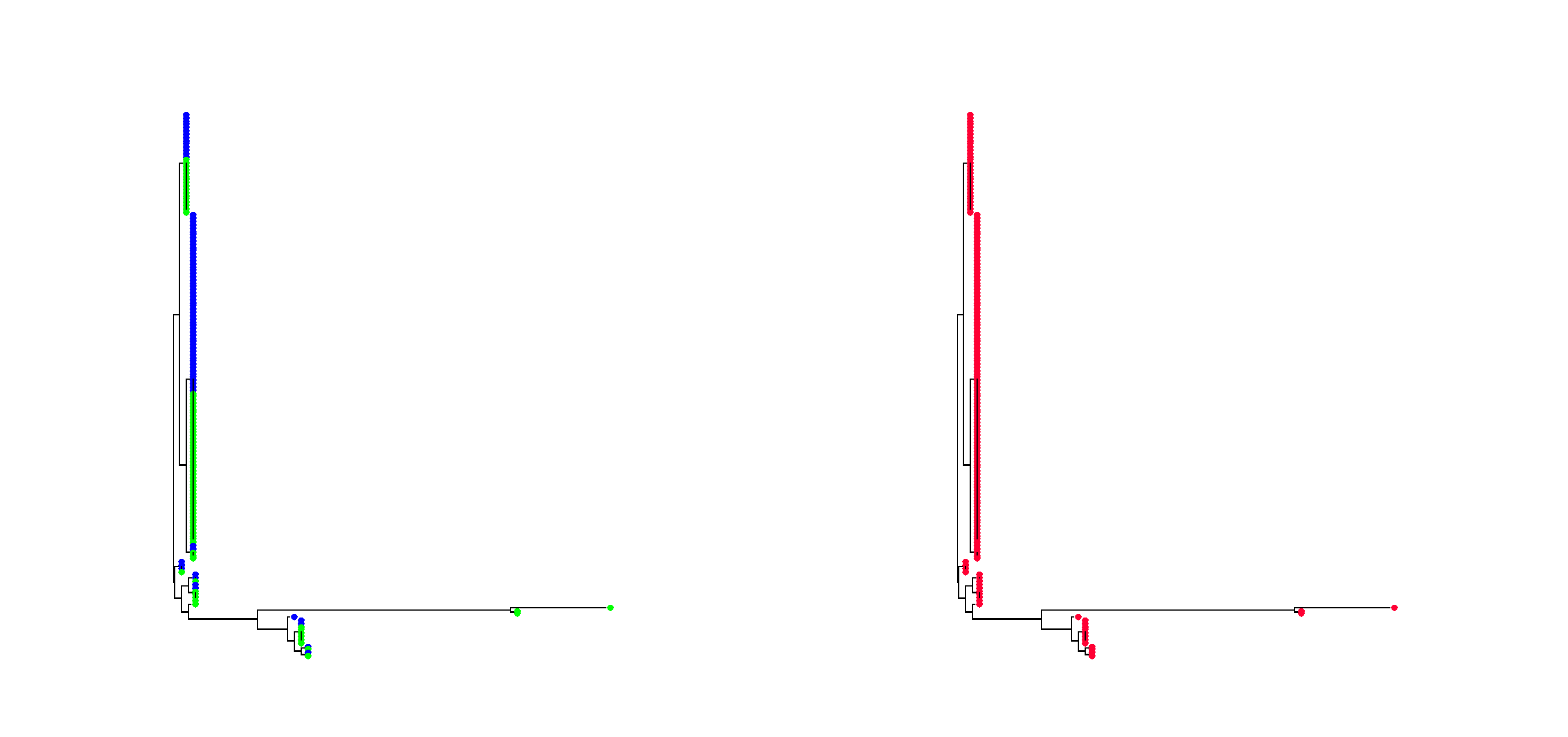

Supplement: Figure S30 — Phylogenetic analysis of Patient 28 Kec haplotypes. The trees in left and right panels are twin trees on which isolation time and tropism, respectively, were mapped. Color codes are as in Figure 2. (TIF) [file pone.0102857.s030.tif]
