# Supplementary material for: HIV-1 Tropism Dynamics and Phylogenetic Analysis from Longitudinal Ultra-Deep Sequencing Data of CCR5- and CXCR4-Using Variants
Source: PLoS One. 2014 Jul 17;9(7):e102857. doi: 10.1371/journal.pone.0102857 (PMC4102574; doi:10.1371/journal.pone.0102857)
Supplement: Table S1 — UDPS results validation by a comparative analysis using conventional cloning. (PDF) [file pone.0102857.s031.pdf]

Pat1 - Sample time A (baseline)

Haplotype	Cloning		UDPS	
	#Clones	RA	#Reads	RA
A	1	5%	30	10%
B	1	5%	11	4%
C	4	21%	55	18%
D	5	26%	44	14%
Others	8	42%	142	47%
Total	19		305	

p* = 0.7170

	#Clones	#Reads
R5	1	67
X4	18	215

p' = 0.0858

Pat3 - Sample time C (month 12)

Haplotype	Cloning		UDPS	
	#Clones	RA	#Reads	RA
A	3	16%	79	21%
B	2	11%	20	5%
C	5	26%	41	11%
D	1	5%	40	11%
E	1	5%	6	2%
Others	7	37%	183	50%
Total	19		369	

p* = 0.2147

	#Clones	#Reads
R5	18	341
X4	1	28

p' = 1.0000

Pat3 - Sample time D (month 60)

Haplotype	Cloning		UDPS	
	#Clones	RA	#Reads	RA
A	4	24%	49	22%
B	1	6%	15	7%
C	1	6%	29	13%
D	3	18%	26	12%
Others	8	47%	107	47%
Total	17		226	

p* = 0.8851

	#Clones	#Reads
R5	15	210
X4	2	16

p' = 0.3646

Pat3 - Sample time E (month 71)

Haplotype	Cloning		UDPS	
	#Clones	RA	#Reads	RA
A	3	21%	147	29%
B	3	21%	86	17%
C	1	7%	6	1%
D	1	7%	14	3%
Others	6	43%	259	51%
Total	14		512	

p* = 0.2706

	#Clones	#Reads
R5	13	465
X4	1	47

p' = 1.000

RA= Relative Abundance

*= Chi Square Test

' = Fisher Exact Test
