# Supplementary material for: HIV-1 Tropism Dynamics and Phylogenetic Analysis from Longitudinal Ultra-Deep Sequencing Data of CCR5- and CXCR4-Using Variants
Source: PLoS One. 2014 Jul 17;9(7):e102857. doi: 10.1371/journal.pone.0102857 (PMC4102574; doi:10.1371/journal.pone.0102857)
Supplement: Table S2 — Laboratory results obtained during the follow-up (expressed as months):predicted HIV tropism (by Geno2pheno); HIV plasma viral load (log copies/mL); CD4 T-cell count (cell/mm3). (PDF) [file pone.0102857.s032.pdf]

Pat 1

	Time							
Tropism	0	12	23	35	44	50	65	77
R5	0.25	-	-	-	-	-	-	-
X4	0.75	-	-	-	-	-	-	-
HIV VL	4.41	<1.7	<1.7	<1.7	<1.7	<1.7	<1.7	<1.7
CD4+	307	17	86	161	202	332	538	400

Pat 5

	Time						
Tropism	0	12	24	48	57	71	83
R5	1.00	-	-	-	-	-	-
X4	0.00	-	-	-	-	-	-
HIV VL	3.80	<1.7	<1.7	<1.7	<1.7	<1.7	<1.7
CD4+	NA	NA	NA	NA	NA	NA	NA

Pat 8

	Time					
Tropism	0	24	34	47	71	83
R5	0.98	-	-	-	-	-
X4	0.02	-	-	-	-	-
HIV VL	4.52	<1.7	<1.7	<1.7	<1.7	<1.7
CD4+	NA	NA	NA	NA	NA	NA

Pat 26

	Time					
Tropism	0	24	48	59	62	71
R5	0.98	-	-	-	-	-
X4	0.02	-	-	-	-	-
HIV VL	4.52	<1.7	<1.7	<1.7	<1.7	<1.7
CD4+	294	357	514	790	722	792

Pat 27

	Time			
Tropism	0	13	49	59
R5	0.04	-	-	-
X4	0.96	-	-	-
HIV VL	5.37	<1.7	<1.7	<1.7
CD4+	143	136	130	149

Pat 28

	Time				
Tropism	0	24	27	41	65
R5	1.00	-	-	-	-
X4	0.00	-	-	-	-
HIV VL	5.37	NA	<1.7	<1.7	<1.7
CD4+	305	256	272	458	479

Pat 3

	Time						
Tropism	0	7	12	25	34	60	71
R5	0.96	0.97	0.95	-	-	0.96	0.96
X4	0.04	0.03	0.05	-	-	0.04	0.04
HIV VL	5.38	5.70	3.57	<1.7	<1.7	3.04	3.56
CD4+	NA	NA	45	397	603	69	68

Pat 6

	Time							
Tropism	-12	0	7	18	20	37	79	91
R5	-	0.97	0.97	-	-	-	0.97	0.99
X4	-	0.03	0.03	-	-	-	0.03	0.01
HIV VL	<1.7	4.10	4.58	2.14	1.78	<1.7	3.15	2.33
CD4+	NA	NA	NA	NA	NA	NA	NA	NA

Pat 7

	Time								
Tropism	0	36	46	57	69	83	93	108	114
R5	0.82	0.97	-	-	-	0.96	0.81	-	-
X4	0.18	0.03	-	-	-	0.04	0.19	-	-
HIV VL	3.59	3.96	<1.7	<1.7	<1.7	4.72	3.73	2.05	<1.7
CD4+	NA	236	263	240	457	388	379	314	NA

Pat 9

	Time				
Tropism	0	10	48	84	95
R5	0.19	-	0.87	0.82	0.72
X4	0.81	-	0.13	0.08	0.28
HIV VL	4.7	3.11	4.48	>5.7	5.67
CD4+	NA	525	NA	91	127

Pat 17

	Time							
Tropism	0	11	36	48	55	62	73	84
R5	0.86	-	-	-	-	-	0.96	-
X4	0.14	-	-	-	-	-	0.04	-
HIV VL	3.94	4.5	4.8	<1.7	<1.7	<1.7	<1.7	<1.7
CD4+	624	699	688	NA	759	NA	1384	1341

Pat 18

	Time						
Tropism	0	24	48	63	73	87	108
R5	0.96	-	-	-	-	-	-
X4	0.04	-	-	-	-	-	-
HIV VL	5.33	<1.7	NA	NA	<1.7	3.55	NA
CD4+	200	306	463	740	619	573	651

Pat 21

	Time							
Tropism	-24	0	12	19	29	37	52	60
R5	-	1.00	-	-	1.00	1.00	-	-
X4	-	0.00	-	-	0.00	0.00	-	-
HIV VL	<1.7	1.98	1.90	<1.7	5.15	4.14	<1.7	<1.7
CD4+	NA	NA	NA	NA	NA	NA	NA	NA

Pat 23

	Time				
Tropism	-36	0	8	23	39
R5	-	0.28	-	-	-
X4	-	0.72	-	-	-
HIV VL	<1.7	5.05	1.90	<1.7	<1.7
CD4+	NA	NA	NA	NA	NA

Pat 24

	Time			
Tropism	0	24	48	54
R5	1.00	1.00	1.00	1.00
X4	0.00	0.00	0.00	0.00
HIV VL	3.96	>5.70	4.00	3.96
CD4+	NA	107	NA	81

Pat 25

	Time								
Tropism	0	8	31	40	53	71	83	88	100
R5	0.62	0.66	0.00	0.58	-	-	-	-	-
X4	0.38	0.34	1.00	0.42	-	-	-	-	-
HIV VL	5.51	5.70	<1.7	<1.7	2.39	<1.7	<1.7	<1.7	<1.7
CD4+	393	448	524	891	NA	485	580	743	858

Pat 13

	Time				
Tropism	0	30	39	53	64
R5	-	1.00	-	1.00	-
X4	-	0.00	-	0.00	-
HIV VL	3.61	2.4	1.90	4.34	1.85
CD4+	292	NA	139	NA	154

Pat 14

	Time						
Tropism	0	9	23	37	45	51	63
R5	-	1.00	-	0.09	0.14	-	-
X4	-	0.00	-	0.91	0.86	-	-
HIV VL	<1.70	<1.70	<1.70	5.46	<1.70	<1.70	<1.70
CD4+	NA	NA	NA	NA	NA	NA	NA

Pat 15

	Time							
Tropism	0	12	27	32	45	54	65	79
R5	-	-	0.96	0.97	0.97	0.97	-	-
X4	-	-	0.04	0.03	0.03	0.03	-	-
HIV VL	6.99	6.57	7.10	6.78	>7.6	>7.6	7.23	6
CD4+	790	1000	532	864	623	628	NA	803

Dash: no data. NA: not available.
